# New Insights into Potential Therapeutic Targets for Neuroendocrine Prostate Cancer: From Bench to Clinic

**DOI:** 10.34133/research.0791

**Published:** 2025-07-31

**Authors:** Maoping Cai, Fengtao Zheng, Yang-Zi Ren, Chuqian Zhen, Dujiang Fu, Xian-Lu Song, Qing Li, Yuanyuan Qu, Zhe-Sheng Chen, Shan-Chao Zhao

**Affiliations:** ^1^ Department of Urology, The Fifth Affiliated Hospital, Southern Medical University, Guangzhou 510630, Guangdong, PR China.; ^2^ Department of Urology, Fudan University Shanghai Cancer Center, Shanghai 200032, PR China.; ^3^ Department of Oncology, Shanghai Medical College, Fudan University, Shanghai 200032, PR China.; ^4^ Shanghai Genitourinary Cancer Institute, Shanghai 200032, PR China.; ^5^ Shantou University Medical College, Shantou 515041, Guangdong, PR China.; ^6^ Department of Oncology, The First Affiliated Hospital of Guangzhou University of Chinese Medicine, Guangzhou 510405, Guangdong, PR China.; ^7^ Department of Urology, Nanfang Hospital, Southern Medical University, Guangzhou 510515, Guangdong, PR China.; ^8^ Department of Radiation and Medical Oncology, Hubei Cancer Clinical Study Center & Hubei Key Laboratory of Tumor Biological Behaviors, Zhongnan Hospital of Wuhan University, Wuhan 430072, China.; ^9^ Department of Radiotherapy, Guangzhou Institute of Cancer Research, the Affiliated Cancer Hospital, Guangzhou Medical University, Guangzhou 510095, Guangdong, PR China.; ^10^ Department of Pharmaceutical Sciences, College of Pharmacy and Health Sciences, St. John’s University, Queens, New York, NY 11439, USA.; ^11^ Department of Urology, The Third Affiliated Hospital, Southern Medical University, Guangzhou 510630, Guangdong, PR China.

## Abstract

Neuroendocrine prostate cancer (NEPC), an aggressive and highly malignant subtype of castration-resistant prostate cancer (CRPC), arises through drug resistance mechanisms involving genomic alterations, epigenetic remodeling, tumor microenvironment (TME) reprogramming, and lineage plasticity. A hallmark of NEPC is its independence from the androgen receptor (AR) pathway, evidenced by diminished or absent AR expression—a key barrier to effective clinical management. Therefore, a comprehensive understanding of NEPC pathogenesis and progression is essential, as it facilitates the identification of potential therapeutic targets and the development of more effective therapies. In this review, we summarize the typical characteristics of NEPC and describe its clinical diagnosis, relevant imaging modalities, current treatment strategies, and associated therapeutic difficulties. We also highlight the core events driving NEPC formation. Furthermore, we discuss potential therapeutic targets for the disease and review pharmacological agents that have demonstrated efficacy against NEPC, aiming to offer innovative perspectives and potential research directions for future NEPC treatment.

## Introduction

Prostate cancer (PCa) is one of the most prevalent male cancers, affecting millions of men worldwide, with a high morbidity and mortality rate [1]. Although androgen deprivation therapy (ADT) is the primary therapeutic choice for advanced PCa and initially creates sensitive responses, patients eventually progress to castration-resistant prostate cancer (CRPC), especially metastatic castration-resistant prostate cancer (mCRPC), within 18 to 36 months [2]. Androgen receptor pathway inhibitors (ARPIs), including abiraterone (ABI), enzalutamide (ENZ), apalutamide (APA), and darolutamide, have been developed for treatment of mCRPC, since the majority of CRPC patients are still AR dependent. Although these treatments show positive outcomes in patients with mCRPC, acquired resistance to ARPIs subsequently emerges and enables tumors to continue to grow [3]. While adenocarcinoma is the predominant histological variant of CRPC (CRPC-adeno), at least 20% to 25% of resistant prostate tumors undergo cellular reprogramming and shed their AR dependence. These tumors, termed neuroendocrine prostate cancer (NEPC), are characterized by the acquisition of a neuroendocrine (NE) phenotype, low or nonexistent AR expression, and independence from AR signaling [4–6].

NEPC is a highly aggressive variant form of PCa, which accounts for approximately 20% of fatal mCRPC [7]. Currently, there are limited definitive and effective treatment options available for NEPC patients, including cytotoxic chemotherapy [8,9]. Even worse, the majority of patients survive less than 1 year after clinical diagnosis of NEPC [10,11]. The origin of NEPC remains elusive, although 2 proposals have emerged: de novo NEPC and treatment-induced NEPC (t-NEPC). Some studies indicate that de novo NEPC may originate from basal cells, luminal cells, or basal cells that acquire luminal traits while losing their basal characteristics [12–14]. Other studies argue that the NEPC may evolve from luminal-like tumor cells called precursor cells in localized NEPC mixed with CRPC-adeno population and advance to pure NEPC [15,16]. As the former has a less than 2% diagnosis rate, the latter is considered the main cause for the formation of NEPC due to its lineage plasticity [17]. Thus, in this review, we focus on t-NEPC.

Unlike typical PCa, levels of prostate-specific antigen (PSA) in NEPC are not elevated, making early diagnosis of NEPC difficult [18]. Recent studies have revealed that the mechanisms underlying treatment-induced NEPC formation are closely related to a variety of factors, such as genomic and epigenetic alterations. In addition, overexpression of oncogenes, inactivation of tumor suppressor genes, imbalance of epigenetic regulatory factors and transcriptional factors, and abnormal expression of receptors on the surface of NEPC cells are closely associated with the occurrence and progression of NEPC [19–32].

Current therapeutic approaches for NEPC primarily extrapolate from successful regimens in small cell lung cancer (SCLC), including platinum-based agents, etoposide, and taxanes administered as monotherapy or in combination. However, these therapies unfortunately lack durability and demonstrate limited therapeutic efficacy [33]. Compared to pure adenocarcinoma cohorts, patients with NEPC exhibit poorer prognosis, demonstrate rapid progression during first-line metastatic therapy, and experience steep declines between subsequent lines of treatment. Following standard therapies, NEPC yields a median progression-free survival (PFS) of 5.2 months and a median overall survival (OS) of 15 months [34]. This may be attributed to several factors: The lack of specific biomarkers [e.g., NE markers such as synaptophysin (SYP), neuron-specific enolase (NSE), chromogranin A (CgA), and neural cell adhesion molecule (CD56)] hinders early detection, diagnosis, and intervention [35–37]; limited therapeutic options with innate or acquired resistance further constrain clinical management [38]; and the immunologically cold tumor microenvironment (TME) characteristic of NEPC substantially compromises immunotherapy efficacy [39].

Emerging treatments focus on combining multiple mechanisms to overcome drug resistance [33]. As our understanding of the genetic, epigenetic, and transcriptional mechanisms regulating development of NEPC continues to advance, increasingly promising therapeutic approaches were developed and applied in preclinical settings, with potential transfer into clinical practice. For instance, using MLN8237 to target inhibition of amplification and up-regulation of proto-oncogenes such as N-MYC and Aurora kinase A (AURKA) can partially impede the progression of PCa and CRPC to NEPC [13,40]. Boosting the expression of tumor suppressors, adjusting epigenetic regulatory factors and transcriptional factors, as well as targeting receptors on the surface of NEPC cells may also play crucial roles in NEPC treatment. It is worth noting that inhibitors targeting these aforementioned targets have the potential to become novel therapeutic drugs or treatments in the future, which is a rare blessing for NEPC patients at present.

To facilitate a comprehensive understanding of potential treatments for NEPC, we provide an overview of the characteristics of NEPC and elucidate its diagnosis, treatment, and remaining problems. We delineate the molecular targets in the development of NEPC and summarize its major origins in this review. In addition, we summarize the potential therapeutic mechanisms and list possible therapeutic agents for NEPC, which may offer novel insights into future treatment approaches.

## NEPC

According to the 2024 Cancer Survey, PCa is one of the most prevalent male cancers [1]. ADT depletes androgen in the endocrine cycle to inhibit its activation of AR signal and prevent the progression of PCa [41]. Although ADT is the primary therapeutic choice for advanced PCa, and patients are originally responsive to ADT, they eventually progress to CRPC, especially mCRPC [2]. Among all types of PCa, CRPC is one of the most common carcinomas in men and affects millions of men worldwide with high morbidity and mortality rates [3]. ARPIs including ABI, ENZ, APA, and darolutamide (DAR) have been developed for treatment of mCRPC, since the majority of CRPC patients are still AR dependent [3]. Treatment with ENZ slows tumor growth but provides limited prevention of tumor recurrence [41]. After treatment with ENZ for a long period of time, it could be observed that the volume of CRPC tumors increased, indicating the formation of tumor resistance to ENZ and tumor recurrence [41]. Additionally, treatment with benzalutamide (BEN) increases the release of BRN4 (Brain4) and BRN2 (Brain2) in extracellular vesicles (EVs) derived from PCa cells, promoting the progression of NEPC [42]. Although treatments of CRPC with more effective AR-targeted therapy (new generation of ARPIs such as ENZ or ABI) prolong the survival of CRPC patients, it may predispose them to progress to NEPC simultaneously [43,44]. Moreover, a growing number of research show that resistance to ADT results in up-regulation of the PKLR/N-MYC/ROMO1 signal, which may drive metabolic reprogramming and reversible cross-differentiation into NEPC [43,45]. Similarly, secondary hormone therapy can increase the likelihood of conversion from CRPC to NEPC as well [27]. Although these treatments show positive outcomes in patients with mCRPC, acquired resistance to ARPIs subsequently emerges and enables tumors to continue to grow [3], and tumors are more likely to undergo conversion from CRPC to NEPC by promoting transcriptional plasticity in intermediate cells [46,47]. In addition to ADT or ARPIs [4], NEPC can arise under radiotherapy [48] or chemotherapy [49]. While adenocarcinoma is the predominant histological variant of CRPC, a quarter of resistant prostate tumors undergo cellular reprogramming and shed their dependence on the AR, thus evading therapies and proliferating [4–6].

### Origin of NEPC

NEPC rarely develops de novo, but it usually occurs after treatment in patients who have already developed CRPC [50]. The development process generally involves either de novo formation from or transformation following CRPC treatment [17,51], and the latter serves as its primary origin [52]. These findings support 2 current theories on the formation of NEPC, namely, de novo NEPC and treatment-induced NEPC. The former suggests that normal prostate epithelial cells may transform into endocrine phenotypes through the deletion of RB1 and the inactivation of p53, which may be the source of de novo NEPC [12]. The latter suggests that refractory PCa with AR loss may transform into NEPC through NE [15]. Given that ADT or ARPIs treatment might induce NE lineage plasticity in prostate adenocarcinoma, the proportion of t-NEPC is extremely higher than de novo NEPC (less than 2%) in clinical observation [17].

The main differences between the 2 are reflected in the following aspects. In terms of cellular origin, de novo NEPC exists at initial diagnosis and arises independently of prior treatment [53]. It may originate from multipotent prostate progenitor cells or develop through transdifferentiation from a common clonal origin with conventional PCa [54]. In contrast, t-NEPC is widely believed to develop through adaptive drug resistance mechanisms following ADT [10] or via epigenetic processes that promote lineage plasticity [53], mediated by transdifferentiation of preexisting adenocarcinoma cells [55]. In aspects of molecular characteristics, de novo NEPC may exhibit distinct RB1 and TP53 alteration patterns compared to t-NEPC [22]. On the contrary, t-NEPC is typically characterized by AURKA and N-MYC amplification/overexpression [56], loss of AR and PSA expression, AR signaling pathway independence [57], frequent co-deletion of RB1 and TP53 [22], up-regulated neuronal transcription factors [Achaete–Scute homolog 1 (ASCL1), neurogenic differentiation 1 (NEUROD1), and POU domain, class 3, transcription factor 2 (POU3F2)] [58], EZH2 overexpression, and DNA methylation alterations [59]. In clinical characteristics, de novo NEPC exhibits an indeterminate clinical course characterized by marked interindividual heterogeneity in treatment responses and clinical outcomes [60]. Notably, most primary NEPC cases present with metastatic lesions at initial diagnosis [61]. In contrast to de novo NEPC, t-NEPC demonstrates intrinsic resistance to AR-targeted therapy [57], with shorter OS typically showing a median survival duration of less than 1 year [62]. Clinically, t-NEPC frequently presents with visceral metastases and is associated with low or undetectable serum PSA levels [10]. These tumors exhibit no response to hormonal therapies [57] but retain partial responsiveness to platinum-based chemotherapy [60,63].

**Fig. 1.**
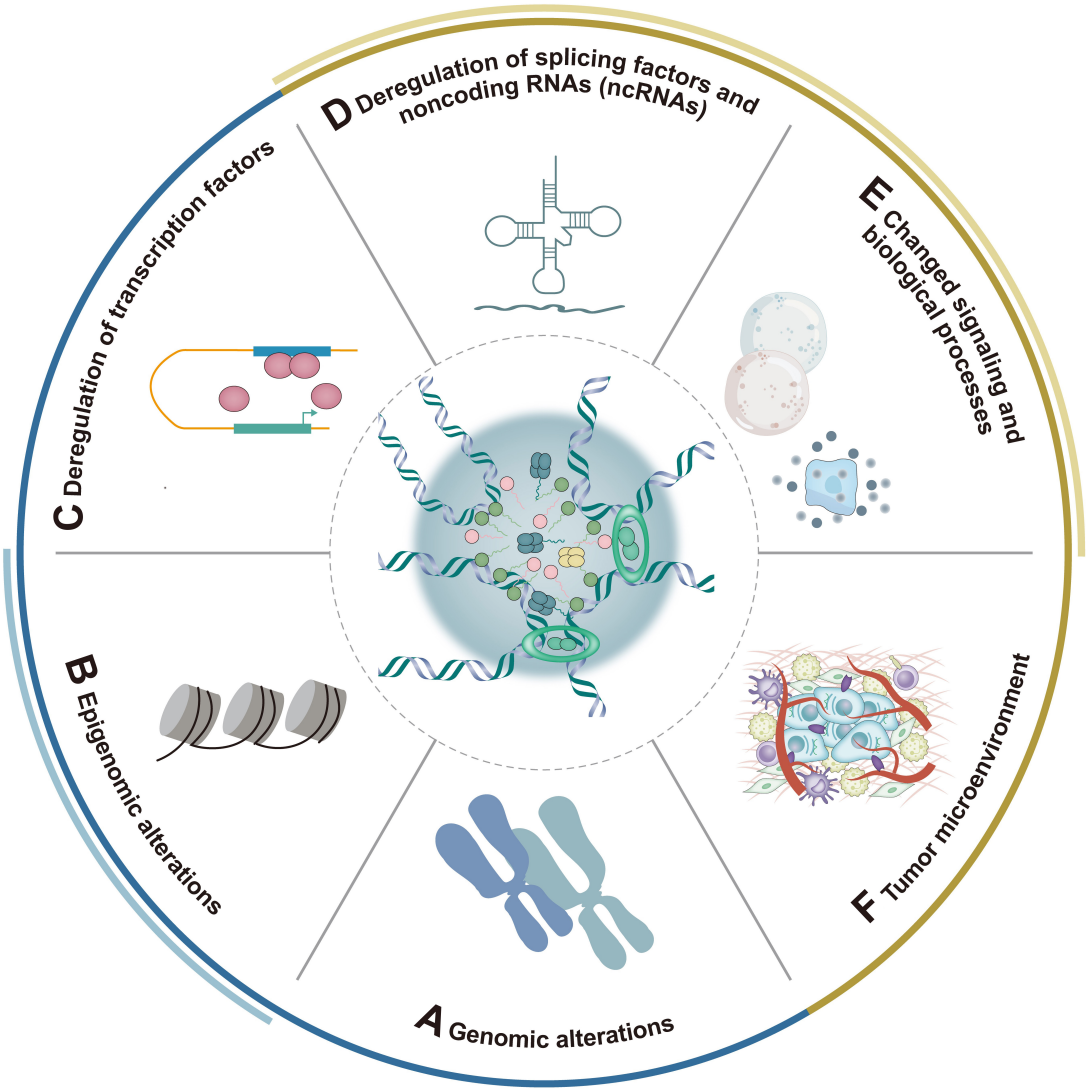
Core events in the transformation from CRPC to NEPC. Multiple factors accounting for the transformation of CRPC to NEPC, including (A) genomic alterations, (B) epigenomic alterations, (C) deregulation of transcription factors, (D) deregulation of splicing factors and noncoding RNAs (ncRNAs), (E) changed signaling and biological processes, and (F) tumor microenvironment (TME).

The precise mechanisms advancing CRPC transformation into NEPC remain largely unknown, and it is still undefined whether NEPC develops through direct transdifferentiation or an intermediate stem-like cell state [64]. However, current research has revealed multiple factors accounting for the transformation of CRPC to NEPC, including: (a) genomic alterations, (b) epigenomic alterations, (c) deregulation of transcription factors, (d) deregulation of splicing factors and noncoding RNAs (ncRNAs), (e) changed signaling and biological processes, and (f) TME [8,65] (Fig. 1). Among these factors, the first 2 have been extensively studied and provide compelling evidence including N-MYC [20,66] and AURKA overexpression [67], as well as PTEN, retinoblastoma-1-encoding gene (RB1), and tumor protein p53 (TP53) inactivation in NEPC [22,68]. The drug resistance related to the development of NEPC coincides to a certain extent with the influencing factors mentioned above for the transition from CRPC to NEPC. There is another key point, namely, lineage plasticity, which means that under therapeutic stress (especially ADT), some prostate adenocarcinoma cells can undergo “lineage transition”, losing the characteristics of adenocarcinoma and acquiring the phenotypes and functions of NE cells. This process is an adaptive drug resistance mechanism to endocrine therapy [69,70].

### Characteristic and classification of NEPC

As a highly aggressive variant form of PCa [71], NEPC is derived from the transdifferentiation of PCa-adeno cells into NE-like cells, which represents the terminal stage of PCa [7,45]. This phenotypic change is described as neuroendocrine differentiation (NED) [72]. NEPC typically emerges late during treatment of CRPC, characterized by small cell morphology, down-regulated AR expression, and up-regulated NE markers [10,45,73], unlike the AR pathway dependence of trend-sensitive PCa or AR mutations or overexpression in CRPC. Besides, there exists a relatively special subtype expressing NE and AR at the same time, which is called amphicrine cancers (AMPC) [74]. The presence of AMPC was confirmed by Aggarwal et al. [52] and Labrecque et al. [75] and at least temporarily responded to the inhibition of the AR pathway because of expression of AR.

Since the vast majority of NEPC expressions are AR deletion/down-regulation and NE up-regulation, and the former is the most important point of drug-resistant mechanism and one of the core characters of NEPC [10], in this review, we mainly discuss the phenotypes in which AR expression is down-regulated or deletion but NE phenotype is up-regulated. Levels of PSA in NEPC are not high [18], and serum NE markers such as CgA, NSE, and gastrin-releasing peptide are enriched [10]. According to the Prostate Cancer Foundation, classification for NEPC consists of (a) usual prostate adenocarcinoma with NE differentiation; (b) adenocarcinoma with Paneth cell NE differentiation (a rare pathological phenomenon primarily characterized by tumor cells possessing unique morphological and immunohistochemical features similar to those of small intestinal Paneth cells; morphologically, these cells exhibit eosinophilic granular cytoplasm, but unlike true Paneth cells, they show negative reactivity for lysozyme); (c) carcinoid tumor; (d) small cell carcinoma; (e) large cell NE carcinoma; and (f) mixed NE carcinoma–acinar adenocarcinoma [76,77]. Of all patients diagnosed with PCa, approximately 80% have local organ-localized disease, 13% to 14% have local metastases, and 6% to 7% have distant metastases [17].

As a late-stage variant of PCa, NEPC is usually accompanied by late-stage manifestations of systemic disease [10]. NEPC patients (45% to 51%) present with metastatic disease at initial diagnosis, and the metastases are usually already locally invasive, such as intestinal, bladder, and kidney involvement, or metastasized to bone and internal organs, such as lungs, liver, and central nervous system [54,78]. Metastases also appeared in the male mammary gland, urinary tract, and adrenal gland [78]. Compared to CRPC, NEPC exhibits aggressive tumor characteristics clinically, including larger tumor size and susceptibility to bone and visceral metastasis [44,73]. Correspondingly, the metastatic pattern of NEPC is also different from that of CRPC. CRPC mostly has bone metastasis, while the incidence of visceral metastasis of NEPC is exceedingly higher than that of other types of PCa [44,73,78]. Its visceral metastasis plays an important role in judging the severity of NEPC. Studies have shown that once NEPC is diagnosed, the number of visceral metastatic organs is one of the most important factors related to survival [10]. Li et al. found that the probability of liver metastasis increased during progression from CRPC to t-NEPC [79]. It can be inferred that the development of conventional prostate adenocarcinoma from hormonal infantilism to CRPC/mixed tumor and then to NEPC is probably a biological continuum. At present, it is only reported that NEPC has a higher probability of visceral metastasis than CRPC, but the specific biological mechanism is not yet clear. In addition, high levels of serum NE markers are characteristic of NEPC [10], which also fails to respond to ADT [73]. Due to its rare and frequently invasive course and its lack of specific diagnosis and monitoring biomarkers, NEPC remains a challenge to diagnose and treat [46].

### Diagnosis of NEPC

Elevated levels of luminal prostate differentiation markers like PSA and prostatic acid phosphatase play a cornerstone role in diagnosis and monitoring of the majority of PCa cases [80]. However, this is not always applicable to NEPC. As a distinct drug-resistant phenotype of mCRPC, NEPC poses challenges in diagnosis [18]. Most NEPC patients exhibit disproportionately low serum PSA levels, much lower than those observed in PCa, and some even show minimal expression [50,81]. Despite variations in NEPC marker expression patterns, all types exhibit strong aggressiveness, with morphology remaining the gold standard for diagnosis [10]. The diagnosis of NEPC typically involves identifying it as a highly invasive subtype of CRPC, which arises from the transdifferentiation process of NED following second-generation anti-androgen therapy targeting the AR. This progression is characterized by rapid advancement [5,42,44,82].

Clinically, NEPC has been suggested to be diagnosed by at least one of the following characteristics: (a) pathological evidence of small cell PCa; (b) exclusively visceral metastases; (c) radiographically predominant lytic bone metastases by plain x-ray or CT scan; (d) bulky (≥5 cm) lymphadenopathy or bulky (≥5 cm) high-grade (Gleason ≥ 8) tumor mass in the prostate/pelvis; (e) low PSA (≤10 ng/ml) at initial presentation (before ADT or at symptomatic progression in the castrate setting) plus high-volume (≥20) bone metastases; (f) presence of NE markers on histology (positive staining of CgA or SYP) or in serum (abnormal high serum levels for CgA or GRP) at initial diagnosis or at progression plus any of the following in the absence of other causes: elevated serum lactate dehydrogenase (LDH) (≥2 × institutional upper limit of normal (IULN)), malignant hypercalcemia, and elevated serum carcinoembryonic antigen (CEA) (≥2 × IULN); and (g) short interval (≤6 months) to androgen-independent progression following the initiation of hormonal therapy with or without the presence of NE markers [63]. At the time of NEPC clinical diagnosis, most patients are already at an advanced stage of NEPC due to its characteristic aggressive nature and rapid progression, as well as its nonspecific symptoms that overlap with other diseases such as hesitancy, dysuria, bone pain, or respiratory symptoms at the site of metastasis [10,11,50]. Regrettably, this may be the dominant reason that most patients survive less than 1 year after clinical diagnosis of NEPC [10,11].

Since NEPC detection is not routinely performed in clinical practice, there is currently no unified guideline on when to perform a biopsy [83]. This directly reflects the value of imaging in the early diagnosis of NEPC. Unfortunately, conventional imaging (CI) such as magnetic resonance (MR), computed tomography (CT), and ultrasound (US) cannot directly distinguish NEPC from other PCa. However, this imaging modality can reflect the presence of aggressive forms. CI only provides indirect information about the presence of necrosis, local invasion, metastasis, or rapid progression [84]. Different from the above CI examinations, at the level of molecular imaging, ^18^F-FDG ([^18^F]-fluorodeoxyglucose, a radiolabeled glucose analog that serves as a tracer for assessing various types of tumors) positron emission tomography/computed tomography (PET/CT) has advantages in NEPC imaging and is a reliable method for identifying aggressive, undifferentiated, or dedifferentiated NED tumors [81,83–87]. Furthermore, high expression of somatostatin receptors (SSTRs) is observed in the majority of patients with NEPC. SSTR-PET/CT imaging using ^68^Ga-DOTATATE or ^68^Ga-DOTANO allows for the specific identification of NEPC lesions, with particularly prominent findings in metastatic sites [81,84,88,89]. In addition to the 2 aforementioned approaches, probes targeting fibroblast activation protein (FAP) also hold promise as a novel adjunctive diagnostic tool for NEPC [90]. On another aspect, multiparametric magnetic resonance imaging (mpMRI) can differentiate prostatic carcinoid (one type of NEPC) from PCa based on the considerably larger size and mild hyperintensity of the tumor on T2W (T2-weighted imaging) images [81], which may improve the detection rate of primary lesions in the prostate and evaluate the aggressiveness of tumors.

Furthermore, the diagnostics of NEPC may benefit from recent advances in liquid biopsy techniques, including circulating tumor DNA (ctDNA) and circulating tumor cells (CTCs) [91], for NEPC detection and disease monitoring. Liquid biopsy offers multiple advantages, including noninvasive sample collection, capacity for serial assessment, and comprehensive insight into tumor biology [91].

Since 2020, blood remains the most extensively studied biofluid, with ctDNA [a special subgroup of cell-free DNA (cfDNA)] representing the most frequently analyzed analyte [92]. A landmark study demonstrated that cfDNA methylation patterns can detect CRPC-NE-associated epigenetic alterations [93]. A targeted panel integrating genomic alterations (TP53, RB1, CYLD protein, AR) and epigenomic features (hypermethylation and hypomethylation at 20 differential loci) applied to ctDNA enables identification of CRPC-NE patients [93]. The analysis of ctDNA facilitates tracking of tumor clonal detection and evolutionary dynamics, potentially identifying high-risk patients prior to histological transformation and paving the way for early intervention strategies [93]. Moreover, research by Fonseca et al. [94] revealed that ctDNA fraction strongly predicts OS, PFS, and treatment response, independent of therapeutic context in mCRPC. Specifically, within an mCRPC cohort encompassing diverse clinical and pathological features (including NEPC subtypes), ctDNA fraction emerged as a robust, independent prognostic biomarker [94].

The prevailing scientific consensus holds that CTCs represent metastatic seeds—rare and heterogeneous yet indicative of cancer progression or dissemination [95–97]. CTCs originates from primary tumors or metastatic sites, which may reflect global tumor characteristics [98]. Combined loss of RB1, TP53, and PTEN in CTCs correlates with lower AR expression and elevated Delta-like ligand 3 (DLL3) expression. Notably, NEPC individuals exhibit higher concordance of alterations among CTCs, suggesting reduced inter-CTC heterogeneity compared to other subtypes [99]. This result aids noninvasive monitoring of NEPC, informs treatment timing, and demonstrates potential for predicting disease progression and therapeutic response. Beltran et al. [99] developed a multivariate CTC classifier utilizing protein expression and morphological variables capable of distinguishing NEPC from CRPC, demonstrating functionality in identifying NEPC-like CTCs. As a minimally invasive approach, CTC analysis enables dynamic monitoring of tumor evolution and may provide utility for early diagnosis of NEPC-associated acquired resistance [98,99]. While liquid biopsy cannot replace traditional histopathological assessment, it represents a promising approach for future NEPC detection strategies.

Similar to the diagnostic modalities discussed above for NEPC, molecular targets [DLL3, tumor-associated calcium signal transducer 2 (Trop2), and carcinoembryonic antigen associated cell adhesion molecule 5 (CEACAM5)] also demonstrate diagnostic utility in both preclinical and clinical settings for this aggressive disease variant [26,27,100]. In preclinical investigations, quantitative polymerase chain reaction (qPCR) and immunohistochemistry (IHC) can be employed to compare the relative levels of AR-regulated markers and the NEPC marker DLL3 in PCa cell lines [101]. Meanwhile, tissue immunofluorescence staining provides direct validation of CEACAM5 expression at the cellular level, while serum CEA testing offers a noninvasive surrogate for monitoring tumor CEACAM5 status [100]. Parallel advancements in surface marker detection employ flow cytometry to quantify Trop2 expression and distinguish molecular subtypes [102,103]. Notably, detection of Trop2 fragments in urine exfoliated cells demonstrated predictive value for aggressive PCa in both mouse models and preliminary clinical samples [104]. Transitioning to clinical applications, molecular imaging innovations show particular promise. The ^89^Zr-labeled DLL3-targeting antibody SC16 (^89^Zr-deferoxamine-SC16) facilitates noninvasive detection of DLL3-positive NEPC lesions via PET imaging [101], while CEACAM5-targeted immuno-PET agents enable in vivo visualization of AR-negative PCa subtypes. Critically, translational validation in advanced mCRPC cohorts confirms serum CEA levels as clinically important correlates of tumor CEACAM5 expression, particularly in NEPC cases [100]. The novel immuno-PET imaging agent ([^68^Ga]Ga-NOTA-T4) achieved noninvasive visualization of Trop2 expression in clinical trials, enabling differentiation between tumors with high versus low Trop2 expression to guide targeted therapy selection [105]. Furthermore, integrated diagnostic strategies combining urinary Trop2 detection with serum NE markers (e.g., CgA) obviously improve early NEPC diagnosis sensitivity [104].

Apart from these, Masone [18] developed a model to assess NEPC risk score, which was found to be extremely higher in NEPC compared to CRPC. This noninvasive method shows promise for diagnosing NEPC but requires further investigation.

### Current treatment for NEPC

Treatment options for NEPC include radiotherapy or chemotherapy alone or in combination. Systemic therapy (primarily platinum-based chemotherapy) remains the mainstay treatment along with palliative local disease management through radiation therapy or surgery depending on individual cases. Among them, the combination of radiotherapy and chemotherapy exhibits the highest survival rate. However, due to the highly invasive nature of NEPC, treatment outcomes are often unsatisfactory [10,11]. It is disheartening to mention that chemotherapy, although somewhat effective in its application, proves inevitably fatal for advanced NEPC patients with a poor prognosis and limited duration of treatment effectiveness [73], further hastening disease progression toward death [50]. In addition, there have been no definitive reports on approved targeted therapeutics demonstrating high efficacy against NEPC, which poses a crucial challenge. In addition to traditional treatment methods, new treatment strategies have brought new hope to patients with NEPC. The latest progress in the field of immunotherapy shows that PD-1 blockade combined with platinum-based chemotherapy may effectively prolong the survival period of patients [106]. Meanwhile, the emergence of protein degradation targeted chimeras (PROTACs) technology has brought a revolutionary breakthrough to cancer treatment [107]. Although most of the current PROTACs research and development focuses on AR reducers, which have limited applicability to NEPC with low AR expression, PROTACs targeting NEPC-specific targets, such as EZH2, have made positive progress in preclinical studies and have shown good application prospects [108]. The current target sites with therapeutic prospects include (a) aberrant expression of surface receptors and proteins including Trop2, neurokinin-1 (NK1R), cholinergic receptor muscarinic 4 (CHRM4), DLL3, and CEACAM5; (b) overexpression of oncogenes N-MYC, AURKA, and mucin 1 C-terminal (MUC1-C); (c) inactivation of tumor suppressor genes RB1, TP53, and PTEN; and (d) imbalance in epigenetic regulatory factors and transcriptional factors such as RE1 silencing transcription factor (REST), forkhead box protein A1 (FOXA1), forkhead box protein A2 (FOXA2), enhancer of zeste homolog 2 (EZH2), SRY-box transcription factor 2 (SOX2), ASCL1, NEUROD1, ONECUT2 (OC2), and ZBTB7A [19–32,41,56,100,109,110].

### Preclinical models of NEPC

Understanding the molecular mechanism of NEPC and evaluating new therapeutic strategies largely rely on preclinical models that can accurately simulate the characteristics of human NEPC. These models successfully reproduced the key pathological features of NEPC, including NED, independence of the AR signaling pathway, and metastatic potential.

In NEPC studies, cell lines derived from patients’ tumors or induced to acquire NED characteristics play an irreplaceable role. Among them, the KUCaP13 cell line originated from a patient with metastatic small cell NEPC who had received LHRH agonists, bicalutamide, and radiotherapy [111]. This cell line has typical NEPC characteristics: absence of AR expression, abnormal Rb pathway, elevated level of NSE, and abnormal expression of NEPC characteristic genes such as AURKA, EZH2, and FOXA2. The LuCaP93 cell line, which also has fundamental research value, originated from primary NEPC patients who received ADT treatment [112]. In addition, classic NEPC cell lines such as NCI-H660 and LASCPC-01 also provide important basis for an in-depth understanding of the pathogenesis of NEPC.

The patient-derived xenograft (PDX) model has become a key platform for NEPC translational research because it can preserve the genetic and phenotypic heterogeneity of the original tumor. These models have demonstrated unique value in multiple research fields: (a) revealing key genes related to tumorigenesis, development, invasion, and metastasis; (b) identifying new therapeutic targets; and (c) evaluating the efficacy of approved drugs and new candidate drugs. For example, the PDX224R-Cx model established by the Melbourne Urology Research Consortium is highly representative. This model originated from a patient with mixed PCa who had both adenocarcinoma and NE phenotypes. The researchers transplanted the tumor tissues under the renal capsule of nonobese diabetic (NOD)/severe combined immunodeficient (SCID) or NOD SCID gamma (NSG) mice that were supplemented with testosterone and castrated. The research results show that the PDX224R model grown in mice supplemented with testosterone maintained mixed pathological characteristics, while the PDX224R-CX model grown in castrated mice exhibited pure NE pathological characteristics [113].

In addition to the abovementioned models, genetically engineered mouse models, as well as emerging technology platforms such as organoids and 3-dimensional culture systems, also demonstrate broad application prospects. These innovative models not only provide new tools for in-depth research on the molecular mechanism of NEPC but also are expected to accelerate the development process of targeted drugs and ultimately improve the clinical treatment effect of NEPC patients. By integrating the advantages of multiple preclinical models, researchers will be able to more comprehensively analyze the complex biological characteristics of NEPC and promote substantive progress in translational medicine research.

Based on these findings, we present a comprehensive list of potential targets both intracellularly and extracellularly, along with the underlying mechanisms responsible for NEPC development. Additionally, we provide an overview of recent experimental studies highlighting promising compounds that exhibit efficacy.

## Potential Targets on the Surface of NEPC Cells

**Fig. 2.**
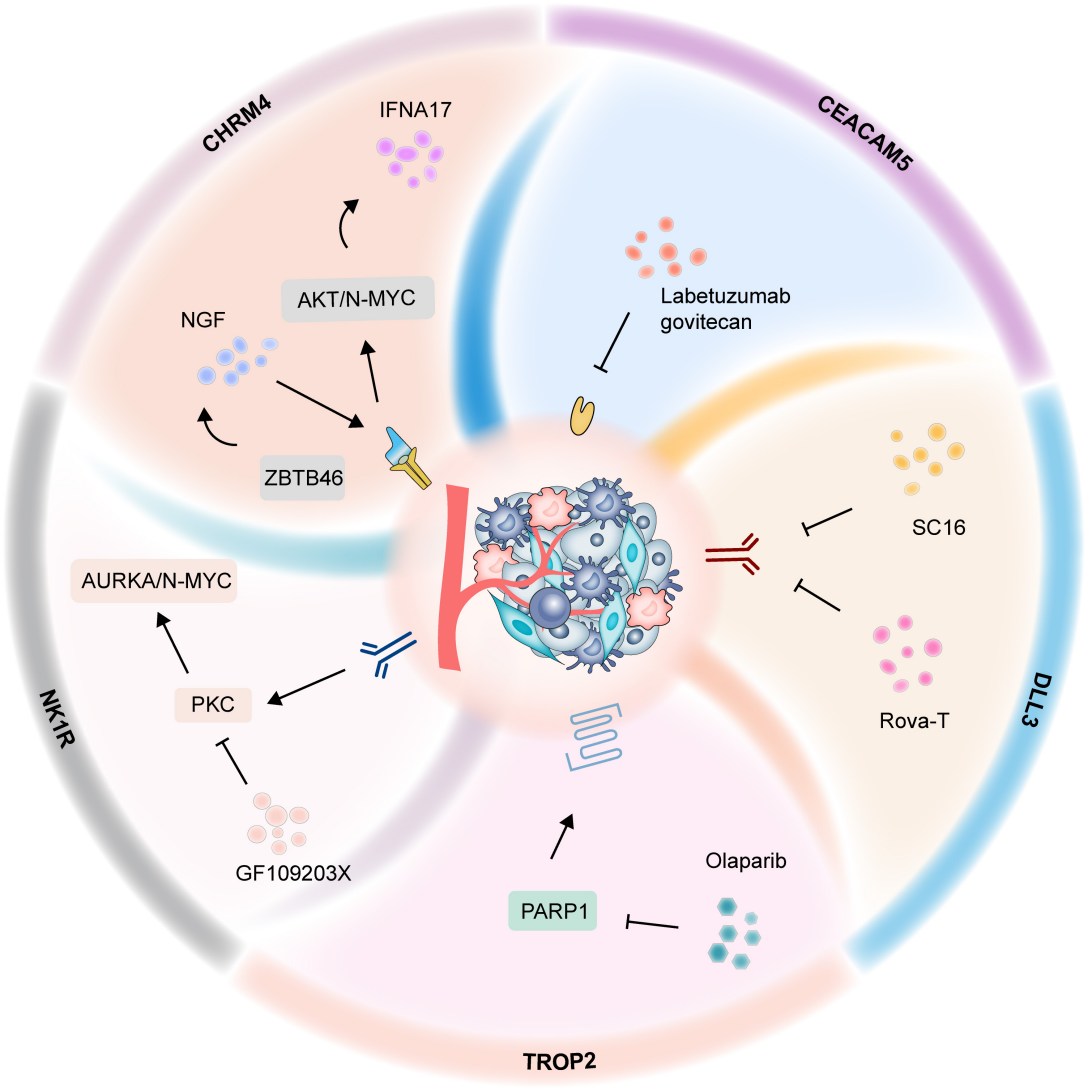
Potential targets on the surface of NEPC cells. Targeted inhibition of PARP1 by OLA may further address Trop2 overexpression, thereby potentially playing a positive role in the suppression of NEPC. NK1R is involved in the PKC/AURKA/N-MYC pathway. The PKC inhibitor GF109203X, through PKC inhibition, evidently reduces AURKA dephosphorylation and induces G2/M cell cycle arrest, consequently inhibiting the development of NED. ZBTB46 up-regulates NGF. Therefore, a potential strategy to modulate NEPC progression and differentiation involves inhibiting NGF, thereby reducing its interaction with CHRM4. The anti-DLL3 antibody SC16 and the anti-DLL3 antibody–drug conjugate Rova-T can inhibit DLL3, thereby exerting an inhibitory effect on NEPC. Labetuzumab govitecan, which targets CEACAM5, has demonstrated therapeutic potential in NEPC cell lines. Trop2, tumor-associated calcium signal transducer 2; NK1R, neurokinin-1; CHRM4, cholinergic receptor muscarinic 4; DLL3, delta-like ligand 3; CEACAM5, carcinoembryonic antigen associated cell adhesion molecule 5; IMMU-132, sacituzumab govitecan; IFNA17, interferon-α17; Rova-T, rovalpituzumab-tesirine; AURKA, Aurora A kinase; MUC1-C, mucin 1 C-terminal; RB1, retinoblastoma-1-encoding gene; TP53, tumor protein p53; REST, RE1 silencing transcription factor; FOXA1, forkhead box protein A1; FOXA2, forkhead box protein A2; EZH2, enhancer of zeste homolog 2; SOX2, SRY-box transcription factor 2; ASCL1, Achaete–Scute homolog 1; OC2, ONECUT2.

At present, aberrant expression of proteins and receptors on the surface of NEPC cells like Trop2 [27], NK1R [29], CHRM4 [28], DLL3 [26], and CEACAM5 [100] makes contributions to driving the development of NEPC. Therefore, we discuss the potential targets of these molecules on the surface of NEPC cells (Fig. 2).

### Trop2

Trop2, a cell surface glycoprotein, plays a pivotal role in promoting cell survival prior to androgen ablation and facilitating lineage plasticity during prostate structural regeneration following androgen supplementation. Trop2 is found to be overexpressed in metastatic and refractory PCa such as NEPC [27]. In CRPC, Trop2 expression persisted in AR^+^/NE^−^, AR^+^/NE^+^, and AR^−^/NE^−^ tumors. Among AR^+^/NE^−^ tumors, 97% express Trop2. Moreover, it is found that Trop2 expression in tumors with liver/lung metastasis is higher than that in tumors with spinal bone metastasis [114]. Numerous studies have reported that approximately 78% of NEPC patients exhibit high levels of Trop2 protein expression [27]. Trop2 functions as a regulator of self-renewal, proliferation, and transformation [115], and the abundant expression of Trop2 markedly enhances tumor growth, metastasis [27,115], and NE phenotype [27]. In addition, 92% of NEPC patients with high Trop2 expression display elevated levels of poly(adenosine diphosphate-ribose) polymerase 1 (PARP1), which mediates reduced DNA condensation and histone methylation in cells overexpressing Trop2 [27]. In this situation, sacituzumab govitecan (IMMU-132), an antibody–drug conjugate consisting of an anti-Trop2 antibody linked to the active topoisomerase inhibitor, represents a cytotoxic drug targeting DNA replication that has shown efficacy against malignancies other than PCa [115].

Trop2-driven NEPC exhibits heightened sensitivity to PARP1 inhibitors, indicating that Trop2 not only acts as a driver of NEPC but also serves as a therapeutic target for treatment of NEPC. Inhibition of PARP1 such as olaparib (OLA) presents a promising strategy for NEPC with overexpressed Trop2, effectively reducing the activation of Trop2 through the proteolytic cleavage pathway. The delayed expression of Trop2 is implicated in tumor growth and metastatic colonization in NEPC via β-integrin and focal adhesion kinase (FAK) signaling, ultimately reversing NE features in vivo [27].

### NK1R

Zhang et al. [116] analyzed publicly available sequencing data from PCa patients and found that the expression of NK1R, a cell surface receptor, was up-regulated in patients with PCa, particularly in cases of NEPC, which exhibited vital stimulation. This suggests a potential association between NK1R and the progression from primary intracavitary adenocarcinoma to NEPC. Elevated levels of NK1R have been clinically correlated with accelerated tumor recurrence and poor survival rate. Moreover, inhibition of AR, one of the treatments for PCa, can enhance the expression of NK1R, thereby stimulating the protein kinase C (PKC)/AURKA/N-MYC pathway in PCa cells. This regulation influences the induction of NE markers and NE-related genes and promotes tumor growth. It means that targeted NK1R therapy can inhibit NE transdifferentiation and tumorigenicity in vivo and in vitro. The NK1R-mediated signaling pathway relies on PKC as a key component. The PKC inhibitor GF109203X could play a key role in reducing AURKA dephosphorylation and inducing cell cycle G2/M arrest, thus restraining the development of NE characteristics and inhibiting tumor growth in vitro [29].

However, a unique case exists within the PKC family, specifically encoded by PRKCI (a style of oncogene), where the protein kinase PKCλ/ι functions as a tumor suppressor in NEPC alongside PTEN. Research has demonstrated that the expression of PKCλ/ι decreases in NEPC, and its loss play pivotal roles in promoting NEPC progression [117]. The down-regulation of PKCλ/ι leads to mTORC1 (mechanistic target of rapamycin complex 1, a key signal complex within cells) activation through reduced lysosomal dispersion and S30 phosphorylation damage, while its interaction with LAMTOR2 [late endosomal/lysosomal adaptor, mitogen-activated protein kinase (MAPK), and MTOR activator 2; a kind of regulator complex protein] drives up-regulated serine biosynthesis via the mTORC1/activating transcription factor 4 (ATF4)/phosphoglycerate dehydrogenase (PHGDH) pathway, and facilitates NEPC differentiation both in vitro and in vivo [117]. Metabolic reprogramming controlled by PKCλ/ι confers a competitive advantage during NEPC differentiation by regulating serine glycine and one-carbon pathway (SGOCP), a crucial metabolic hub for proliferation and an epigenetic control factor for gene expression that promotes intracellular S-adenosyl methionine (SAM) biosynthesis [117]. In conclusion, inhibition of the downstream mTORC1/ATF4/SGOCP of PKCλ/ι exerts a positive impact on NEPC [117].

### CHRM4

CHRM4, a G protein-coupled receptor, is highly expressed in PCa cells following ADT [28]. Abundant activation of the CHRM4-driven AKT/N-MYC pathway up-regulates interferon-α17 (IFNA17) in the TME to promote NED of PCa [28]. Therefore, there is no doubt that small compounds targeting CHRM4 possess the potential to effectively suppress NED and tumor growth in NEPC.

Nerve growth factor (NGF) contributes to the progression of malignancy and regulates NEPC differentiation through physical interaction with CHRM4 [47]. Simultaneously, ZBTB46, an ADT-stimulated transcription factor associated with NEPC polarization, up-regulates NGF in NEPC [47]. This pathway suggests that targeting NGF-CHRM4 signals or ZBTB46-mediated transcription could be a potential approach for treating or inhibiting NEPC [47,118]. Activated NGF up-regulates CHRM4 and connects AKT signaling activation with N-MYC stimulation, which enhances NEPC reprogramming. In addition, NGF stimulates p38-MAPK to activate downstream NTRK1 and promote cell migration, invasion, and metastasis [118,119]. Pharmacologically, a block or knockdown of NGF substantially inhibits ADT resistance, CHRM4-mediated NEPC differentiation, and AKT-N-MYC signaling activation [118], thus impeding the progression of NEPC.

### DLL3

DLL3, a distinctive protein present exclusively on the surface of NEPC cells, is absent on the surface of normal cells [71]. DLL3 is highly expressed in solid tumors, especially NEPC [26], affecting approximately 76.6% of NEPC patients [120]. DLL3 is highly expressed in solid tumors, especially NEPC [26]. Puca et al. [120] evaluated 47 CRPC-NE samples and found that DLL3 was expressed in 36 samples (76.6%). DLL3 could be detected in a small number of CRPC-adeno tumors (12.5%), but not detected in benign prostate or localized PCa. Similarly, in CRPC-NE cases analyzed by RNA sequencing (RNA-seq) and NanoString, 73.68% and 71.10% of the cases showed high DLL3 mRNA expression, and 10.91% (RNA-seq) as well as 12.50% (NanoString) of CRPC-adeno samples also showed high DLL3 mRNA expression [120]. Korsen et al. [71] developed a potential treatment for NEPC by using anti-DLL3 antibody SC16 radiolabeled with the β-emitting radioisotope lutetium-177, also known as a molecularly targeted radiotherapeutic approach. Additionally, rovalpituzumab-tesirine (Rova-T), a first-in-class antibody–drug conjugate that targets DLL3 to deliver a cytotoxic compound directly to NEPC tumor cells [26], and specific T cell contact antibodies (NCT03319940 and NCT04471727) serve similar role as DLL3 inhibitor, which might reduce the amount of DLL3 on the surface of NEPC cells [109], and inhibit the growth of NEPC. In addition, tarlatamab (AMG 757), which targets DLL3 and CD3 molecules on the surface of T cells, is a bispecific antibody prepared based on the next-generation bispecific T cell agonist (HLE BiTE) technology. By recognizing and ligating CD3 (expressed on the cell membrane of T cells) and DLL3 (expressed on the cell membrane of NET), it promotes T cells to recognize NET cells and activates T cell-mediated tumor lysis [121]. Currently, AMG 757 has been reported to have a certain remission effect in SCLC, and the next clinical trial will be conducted in NEPC patients in the future.

### CEACAM5

CEACAM5, a cell surface protein, belongs to the CEA family and is highly expressed in NEPC [100]. Labetuzumab govitecan, a drug targeting CEACAM5, has demonstrated potent antitumor activity in preclinical models of CRPC (including NEPC). Its mechanism of action involves DNA damage and exerts positive therapeutic effects [100]. Engineered chimeric antigen receptor T (CAR-T) cells targeting CEACAM5 exhibit therapeutic potential by inducing antigen-specific cytotoxicity in NEPC cell lines [122].

## Promising Targets in NEPC Cells

NEPC is characterized by absent or minimal AR expression and lacks activation of canonical AR downstream signaling pathways (including PSA production), rendering it intrinsically resistant to conventional ADT and second-generation ARPIs such as ENZ and ABI [75,123,124]. This therapeutic resistance arises not only from intrinsic molecular heterogeneity but also through dynamic lineage plasticity mechanisms. Specifically, ADT-mediated AR signaling suppression triggers a phenotypic switch to AR-independent states via activation of NE transcriptional programs involving master regulators like BRN2 and ASCL1 [64].

The current standard of care for NEPC relies on platinum-based chemotherapy (typically cisplatin combined with etoposide); however, outcomes remain poor, with a median OS of approximately 7 months [10]. This limited efficacy stems from multiple resistance mechanisms: (a) EZH2-mediated epigenetic silencing of tumor suppressors (e.g., RB1) promotes chemoresistance [13], and (b) cancer stem cell populations maintain treatment-refractory properties through sustained Wnt/β-catenin pathway activation [125]. These molecular insights highlight the critical need to develop novel targeted therapeutic strategies that address the unique biology of NEPC beyond conventional cytotoxic approaches.

Carcinogenic factors N-MYC, AURKA [19,56], and MUC1-C [21], loss of tumor suppressors PTEN, RB1, and TP53 [22], and amplification of epigenetic controls and transcriptional factors like FOXA1 [23], FOXA2 [24], EZH2 [20], REST [30], and BRN2 [41] have the potential to drive PCa/CRPC transformation into NEPC.

**Fig. 3.**
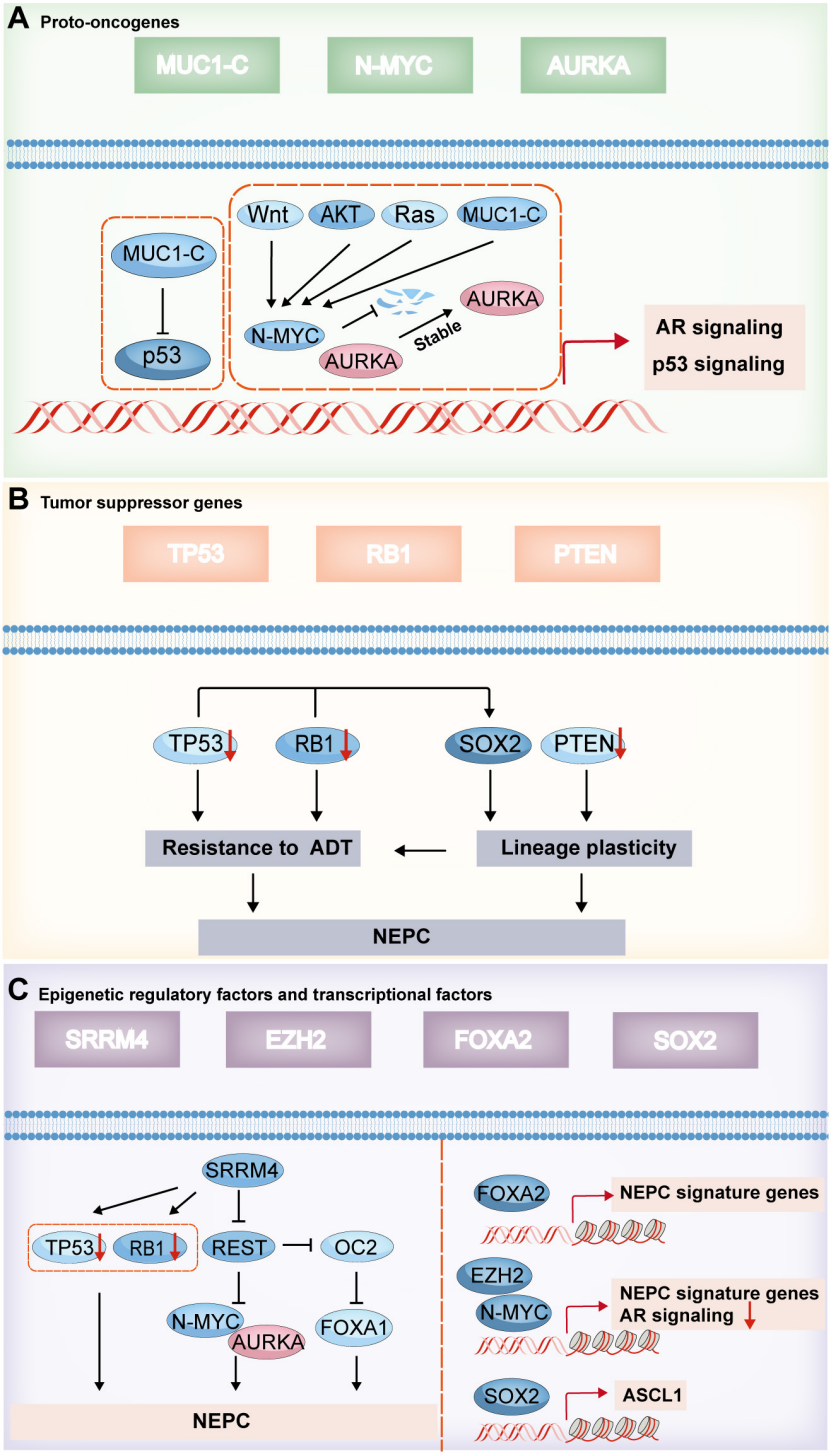
Potential targets of cell surface receptors on NEPC formation. (A) Proto-oncogenes: Key proto-oncogenes including MUC1-C, N-MYC, and AURKA are stabilized by upstream signals (Wnt, AKT, Ras, and MUC1-C), thus influencing AR and p53 signaling pathways. (B) Tumor suppressor genes: Loss or down-regulation of tumor suppressor genes (TP53, RB1, and PTEN) promotes resistance to ADT and lineage plasticity, contributing to NEPC development. SOX2 is also involved in promoting lineage plasticity. (C) Epigenetic regulatory factors and transcriptional factors: Factors such as SRRM4, EZH2, FOXA2, and SOX2 regulate NEPC progression. SRRM4 suppresses TP53 and RB1, leading to downstream effects on REST, N-MYC, AURKA, and FOXA1. EZH2, FOXA2, and SOX2 regulate NEPC signature genes and AR signaling, with SOX2 also promoting ASCL1 expression. The interplay of these factors drives the transcriptional reprogramming and epigenetic regulation characteristic of NEPC.

Herein, we delineate the amplification and up-regulation of oncogenes, the inactivation of tumor suppressors and the imbalance of transcription factors, and the epigenetic regulatory factors related to the formation of NEPC (Fig. 3). Based on these, we further summarize the current treatment methods that play potential roles in targeting inhibition of NEPC (Table 1).

**Table 1.** Promising targets of NEPC

Target				Related Drug/Compounds	Function	References
Proto-oncogenes	MYC and AURKA	AURKA		CD532	Inhibit kinase activity, protein interaction disruption and conformational disruption	[19]
				VX680	Inhibit kinase activity	[195]
				Icaritin	Inhibit kinase activity	[196]
			AURKA-N-MYC	Alisertib (MLN8237)	Inhibit kinase activity	[13,40]
			AURKA-N-MYC	MLN8054	Inhibit kinase activity	[20]
			AURKA-N-MYC/MAX	PHA680626	Inhibit kinase activity and conformational disruption	[129]
			AURKA- N-MYC -PARP-DDR	Danusertib (PHA739358)	Inhibit kinase activity	[129]
			AURKA-N-MYC	Compound 70812	Inhibit kinase activity and protein interaction disruption	[19]
		MYC		Compound MYCi361	Inhibit protein–protein interactions	[197]
				Compound MYCi975	Inhibit protein–protein interactions	[196]
		N-MYC	AURKA-N-MYC	CD532	Protein interaction disruption and conformational disruption	[19]
			PI3K-AKT-mTOR	BKM120	Inhibit the activity of PI3K	[20,54]
				BEZ235	Inhibit the activity of PI3K and mTOR	[20,54]
				PX-866	Inhibit the activity of PI3K	[198]
				RAD001	Inhibit the activity of mTOR	[20]
				MK2206	Inhibit the activity of AKT	[20]
				LY294002	Inhibit the activity of PI3K	[151,199]
				Ipatasertib	Inhibit the activity of AKT	[20,200]
			PKLR-N-MYC-ROMO1	Inhibitor of BET family (ZEN-3694, JQ1, OTX-15)	Suppress transcriptional activity	[43,201,202]
		MYC/MAX	MYC/MAX-DNA	Compound EN4	Suppress transcriptional activity	[203,204]
			MYC/MAX-DNA	Small molecule KJ-PYR-9	Suppress transcriptional activity	[205]
		N-MYC/MAX	N-MYC/MAX-DNA	Compound 70812	Suppress transcriptional activity and inhibit AURKA	[19]
			N-MYC/MAX-DNA	Compound 70551	Suppress transcriptional activity	[19,66]
			N-MYC/MAX-DNA	Compound VPC-70619	Suppress transcriptional activity	[204]
		C-MYC/MAX	C-MYC/MAX-DNA	Small molecule L755507	Suppress transcriptional activity	[206]
		ATM	N-MYC-ATM	Ku60019	Inhibit kinase activity	[156]
		ALK	ALK-Wnt-β-Catenin	Alectinib	Inhibit kinase activity	[125]
		AKT	N-MYC-FKBP5-PHIPP-AKT	Fluoxetine	Inhibit kinase activity	[155]
			ROS-N-MYC-AKT	Fludarabine phosphate	Inhibit kinase activity	[44]
		PKC	PKC-AURKA-N-MYC	GF109203X	Inhibit kinase activity	[29]
				Enzastaurin	Inhibit kinase activity	[207]
	MUC1C	MUC1C	MUC1C-MYC-BRN2-SOX2	GO-201	Inhibit protein–protein interactions	[21,208,209]
			MUC1C-MYC-BRN2-SOX2	GO-203	Inhibit protein–protein interactions	
Tumor suppressor	RB1	GPX4	GPX4-ACSL4		Inhibit	[140]
		RB1		Dinaciclib	Inhibit the activity of CDK	[145]
	TP53	LSD1	LSD1-TP53	SP-2509	Inhibit LSD1	[143]
				SP-2577	Inhibit LSD1	[143,210]
				CC-90011	Inhibit LSD1	[211]
	PEG10	A special shRNA	shRNA-PEG10		Inhibit	[17]
		TGF-β	PEG10-TGF-β	LY2157299 (Galunisertib)	Inhibit kinase activity	[212]
				LY364947	Inhibit kinase activity	[213]
	TRIM36	HK2	HK2-TRIM36	3-bromopyruvate	Inhibit kinase activity	[213]
		Glycolysis	HK2-TRIM36-Glycolysis	2-deoxy-d-glucose	Induce the degradation of TRIM36 protein	[213]
			IL-6-STAT3	Ketotifen	Target the IL-6/STAT3 signaling pathway	[214]
				LLL12	Target the IL-6/STAT3 signaling pathway	[215]
				P6	Target the IL-6/STAT3 signaling pathway	[215]
				CNTO 328	Target the IL-6/STAT3 signaling pathway	[215,216]
				Gallellalactone	Target the IL-6/STAT3 signaling pathway	[217]
Epigenetic regulatory factors and transcriptional factors	SRRM4	Bif-1	SRRM4-Bif-1-anti-apoptosis		Inhibit	[218]
		GIT1-A	SRRM4-GIT1-A		Inhibit	[150]
		LSD1+8a	SRRM4-LSD+8a		Inhibit	[219]
		MyD88	SRRM4-BHC80-2-MyD88-P38-TTP		Inhibit	[197]
		MEAF6-1	SRRM4-MEAF6-1		Inhibit	[161]
	REST	PI3K-AKT	PI3K-AKT-REST		Activate	[151]
			GPK3-HDAC2-TSP1-REST		Inhibit	[220]
		SPINK1	REST -SPINK 1		Inhibit	[221]
		SK1	SK1-MEPK-REST	FTY720	Inhibit kinase activity	[222,223]
		HP1α	HP1α -REST		Inhibit	[224]
		LncRNA-p21	AR-LncRNA-p21-EZH2-STAT3	DZNEP	Inhibit the histone methyltransferase activity of EZH2 and interfere with the LncRNA-p21-EZH2-STAT3 signal axis	[154,225]
	EZH2	LncRNA-p21	AR-LncRNA-p21-EZH2-STAT3	GSK126	Inhibit the activity of EZH2 and interfere with the LncRNA-p21-EZH2-STAT3 signal axis	[154,225]
		CDK1	CDK1-EZH2	Docetaxel	Interfere with the cell cycle process regulated by CDK1	[153]
		TCF4	Wnt-β-catenin -TCF4-EZH2	LGK974	Inhibit the protein stability of TCF4 and interfere with the Wnt-TCF4-EZH2 signal axis	[125,153,226]
				XAV-939	Inhibit protein–protein interactions and inhibit the activity of the Wnt/β-catenin signaling pathway	[125]
				ICG-001	Inhibit the activity of the Wnt/β-catenin signaling pathway	[125]
		PACGAP1	E2F1-PACGAP1-EZH2-N-MYC		Inhibit	[227]
		EHF	EHF-EZH2		Activate	[228]
		TSP1	TSP1-EZH2	TSA	Suppress transcriptional activity	[225]
		PKA	PKA-CREB	Propranolol	Inhibit kinase activity	[225]
		CREB	CREB-TSP1-EZH2		Inhibit	[225]
		EZH2	EZH2-H3K27 methylation	GSK503	Inhibit the enzymatic activity of EZH2	[20,111,225,227]
				GSK343	Inhibit the enzymatic activity of EZH2	[20,229]
				GSK126	Inhibit the enzymatic activity of EZH2	[20,123]
				EPZ6438 (Tazemetostat)	Inhibit the enzymatic activity of EZH2	[20,111,153,225,226]
	FOXA1	MiR-32			Inhibit	[230]
		MiR-147b			Inhibit	[231]
		MiR-194	MiR-194-FOXA1		Inhibit	[157]
			FOXA1-IL-8-MAPK-EPK-ENO2		Inhibit	[159]
	FOXA2	LINC00261	LINC00261-SMAD2/3-FOXA2		Inhibit	[232]
		PHF8	PHF8-FOXA2		Inhibit	[233]
		KIT	FOXA2-KIT	Dovitinib	Inhibit kinase activity	[24,160,234]
				Imatinib	Inhibit kinase activity	[24,160]
				Sunltinib	Inhibit kinase activity	
				Sorafenib	Inhibit kinase activity	
				Cabozantinib	Inhibit kinase activity	[24,160,235–237]
		SIAH2	SIAH2-HIF-FOXA2	Menadione	Inhibit SIAH2	[238]
				RLS-24	Inhibit SIAH2	[239]
		HIF-1α		TH-302	Reduce the induction of HIF-1α and affect the stability and activity of HIF-1α	[165]
		SRC	SRC family kinases signaling	Dasatinib	Inhibit kinase activity	[240–243]
			MEK-ERK	TMT212	Induce the degradation of MEK	[244]
				SCH772984	Inhibit the kinase activity of ERK	[244]
	FOXC2	P38-MAPK		SB203580	Inhibit kinase activity	[180,212]
	SOX2	LIN288	LIN288-Let-7-SOX2	Ln7	Prevent the binding of LIN28B to the pre-miRNA of Let-7	[245,246]
				Ln15		
				Ln115		
		NRP2	NRP2-VEGFR2-STAT3-SOX2		Inhibit	[247]
		SRRM4	SRRM4-SOX2		Inhibit	[138]
		TRIM59	TRIM59-SOX2		Inhibit	[147]
			TBX2-MiR-200c-3p-SOX2-N-MYC		Inhibit	[248]
		LSD1	SOX2-LSD1		Inhibit	[162]
				CC-90011	Inhibit the demethylase activity of LSD1	[211]
		H19	SOX2-H19		Inhibit	[249]
	OC2		OC2-SRRM4	CSRM617	Eliminate the OC2 protein	[167]
	ZBTB7A	RET		Cabozantinib	Inhibit kinase activity	[24,160,235–237]
				LOXO-292		[169]
				BLU-667		[169]
				AD80		[169]
	ZBTB46	LIF	ZBTB46-LIF	EC330	Degrade LIF protein	[250]
		PTGS1	ZBTB46-PTGS2	NS-398	Bind and destroy the PTGS2 protein	[251]
	ASCL1	DLL3	ASCL1 -DLL3	Rpcalpituzumab Tesirine	Antibody-mediated cytotoxicity	[120,163]
		ASCL1	ASCL1-CREB-Ferroptosis Resistance		Inhibit	[25]
	NEUROD1				Inhibit	[109,164]
Others	CBX2	CBX2		SW2_152F	Inhibit protein-histone interaction	[252]
	DRD2	DRD2		ONC-201	Block receptor	[170]
	NSD2	NSD2		MCTP-39	Inhibit the activity of histone methyltransferase	[171,253]
	SWI/SNF complexes	SWI/SNF complexes			Inhibit	[172]
	ApoA-I	ApoA-I			Inhibit	[173]
	SNAI1	SNAI1		NPI-0052 (Marizomib)	Inhibit the proteasome and indirectly affect SNAI1	[254,255]
	SNAI2	SNAI2		MLN4924 (Pevonedistat)	Inhibit Neddylation	[256]
	GGPS	GGPS		Digeranyl Bisphosphonate	Inhibit GGPS	[257]
	DNMT	DNMT		Decitabine	Inhibit the activity of DNA methyltransferase	[117]
				Azacytidine		[258,259]
	DEK	DEK		DEK-targeted aptamers	Bind to and interfere with DEK protein	[260,261]

PKLR, pyruvate kinase L/R; ROMO1, reactive oxygen species modulator 1; ATM, ataxia telangiectasia mutated kinase; ALK, anaplastic lymphoma kinase; MEAF6-1, MEAF MYST/Esa1-associated factor 6-1; SK1, sphingosine kinase 1; HK2, hexokinase 2; TRIM36, trigred motif 36; HDAC2, histone deacetylase 2; TSP1, thromboplastin 1; SPINK1, serine peptidase inhibitor, kazal type 1; EHF, a member of epithelial-specific ETS transcription factor family; PACGAP1, a member of the GAP enzyme activating protein family; TCF4, transcription factor 4; LncRNA-p21, long noncoding RNA-p21; MiR-194, microRNA-194; ENO2, enolase 2; LINC00261, long intergenic nonprotein coding RNA 261; PHF8, plant homeodomain finger-containing protein 8; NRP2, NEUROPILIN 2; TRIM59, a carcinogenic protein of TRIM family; HIF-1α, hypoxia inducible factor 1α; MAPK, mitogen-activated protein kinase; H19, a long-stranded noncoding RNA; LIF, leukemia inhibitory factor; IMMU-132, sacituzumab govitecan; IFNA17, interferon-α17; DRD2, dopamine receptor D2; ApoA-I, apolipoprotein A-I; NSD2, nuclear receptor binding SET domain protein 2; SNAI1, snail family transcriptional repressors 1; SNAI2, snail family transcriptional repressors 2; GGPS, geranylgeranyl pyrophosphate synthase; DNMT, DNA methyltransferase; NEUROD1, neurogenic differentiation 1

### Proto-oncogenes

#### MYC and AURKA

N-MYC and AURKA can be detected simultaneously in 40% of NEPC, and they synergistically induce NEPC [56]. The actual physical protein–protein interaction between N-MYC and AURKA forms a complex that serves to shield N-MYC from proteasome degradation and enhance the stability of the AURKA protein [19,40]. This interaction exacerbates downstream carcinogenic effects and NED in PCa [19].

Numerous studies have consistently demonstrated the overexpression of the carcinogenic transcription factor N-MYC and cell cycle kinase AURKA in the majority of highly proliferative and invasive metastatic NEPC [20,43,56]. Dysfunctional MYC genes, including C-MYC and N-MYC, which are pivotal regulators of cellular proliferation [126], have been identified in approximately 70% of human cancers [127]. These genes serve as master regulators of MYC family transcription, activating genes that influence cancer hallmarks such as sustained proliferation and growth [66]. Extensive research has revealed that N-MYC plays a crucial role as an oncoprotein essential for NE tumors and promoting tumor invasiveness in PCa [19,51]. N-MYC expression can be detected in 40% of NEPC, while N-MYC amplification is not observed in benign prostate [56]. Furthermore, carcinogenic signaling pathways like Wnt, Ras, and phosphatidylinositol 3-kinase (PI3K)/AKT can also enhance their tumor-promoting function by reinforcing the stability of N-MYC [20,128]. The PARP1 inhibitor OLA specifically targets the N-MYCPARP-DNA damage response (PARP-DDR) pathway, generating therapeutic effects such as decreased cell viability, colony formation, and migration, similar to PARP1 knockout observed in vitro. Furthermore, it remarkably inhibits the growth of NEPC in NCI-H660 and PDX 144-13C subcutaneous models in vivo [129]. A study combining PARP inhibitors (PARPi; OLA) with cyclin-dependent kinase 4/6 inhibition (CDK4/6i; palbociclib or abemaciclib) shows the p-RB1–E2F1 signal axis to be synergistically suppressed at both the transcriptional and posttranslational levels [130]. This further disrupts cell cycle progression and inhibits E2F1 gene targets, leading to increased apoptosis both in vitro and in vivo. Moreover, the combined effect of these 2 treatments is superior to the effect of either treatment alone [131]. Regrettably, this combined treatment approach has not yet been translated into specific clinical trials for NEPC. Additionally, talazoparib (TALA) plays an analogous role in reversing NE phenotype [27].

AURKA is a serine/threonine kinase involved in the formation of the mitotic spindle, separation of centrosome, and the G2-M transition during cell cycle, with potential oncogenic properties [56]. In NEPC, over 50% of tumor cells exhibit robust cytoplasmic expression of AURKA [56], which plays a crucial role in the NED of PCa [67]. Down-regulation of NKX3.1, a prostate-specific tumor suppressor, mediated by AURKA, is the primary mechanism driving CRPC to differentiate into NEPC [132]. Additionally, there are reports indicating that targeting AURKA effectively inhibits PCa cell proliferation driven by CXCR7, an atypical chemokine receptor up-regulated in PCa following treatment with ADT or ARPIs [133].

#### MUC1-C

Mucin 1 (MUC1) is a heterodimer protein that is aberrantly overexpressed in advanced NEPC and serves as a potential therapeutic target. It is closely related to the characteristics of cancer cells [21,134]. MUC1 encodes a polypeptide that undergoes autocleavage into N-terminal (MUC1-N) and C-terminal (MUC1-C) subunits [135]. Furthermore, MUC1-C actively participates in inducing the NE process of N-MYC, EZH2, and PCa related to the progression of NEPC by inhibiting the p53 pathway and activating MYC -Brn2 trail [21,136]. In recent years, Yasumizu et al. [21] found that targeting MUC1-C in vitro and in PCa tumor xenograft models restricted BRN2 transcription as well as NED induction, self-renewal capacity enhancement, and oncogenicity, such as GO-201 and GO-203. In addition, during early-phase clinical trial MULTIs, GO-203 was evaluated for its acceptable safety profile and for evidence of antitumor activity [137]. It is currently under further development to target cancers specifically expressing MUC1-C, such as NEPC [21]. Undoubtedly, MUC1-C represents a promising target for curbing NEPC.

### Tumor suppressor

The deletion of PTEN, RB1, and TP53 is exceptionally prevalent in NEPC due to their pivotal roles in NEPC development [22]. Specifically, the concurrent deletion of RB1 and PTEN enhances lineage plasticity and trans-differentiation potential, while the cancellation of RB1 and TP53 confers resistance to ADT [68]. To put it simply, the combined effect of deletion of these 3 genes along with AR inhibition promotes the initiation and progression of NEPC [138].

RB1 loss is observed in approximately 70% of NEPC and in 10% to 30% of CRPC [139]. The RB1 gene functions as a tumor suppressor by inhibiting the G1/S transition through its interaction with E2F family transcription factors, which are commonly deleted in NEPC. This interaction maintains the E2F family transcription factors in an inactive state at the transcriptional level [120]. Loss of RB1 function results in dysregulation of gene networks associated with E2F and has been implicated in driving tumorigenesis and NED progression [140,141]. In RB1-deficient NEPC tumors, up-regulation of ACSL4 (a key enzyme promoting lipid peroxidation and triggering ferroptosis) renders cancer cells susceptible to ferroptosis inducers. However, tumor cells overexpress GPX4 (an antioxidant enzyme) to regulate ferroptosis [140]. Based on this, GPX4 inhibitors can effectively suppress the overexpression of GPX4 in tumor cells, thus reactivating ferroptosis and impeding tumor growth. The up-regulation of ACSL4 expression and enhancement of lipid peroxidation rate induced by RB1 loss further intensify cancer cell dependence on GPX4 for iron homeostasis blockade. Frustratingly, this renders cancer cells more susceptible to GPX4 inhibitors or general inducers of iron decay [140].

Deficiency of TP53 confers resistance to anti-androgen therapy [123]. TP53 buildup is detected in 56% of small cell neuroendocrine prostate cancer (SCNPC), while TP53 mutations are present in 60% of SCNPC [142]. Lysine-specific demethylase 1 (LSD1), a histone demethylase, can impede the downstream pathway of TP53 by reducing its occupancy on the target gene, which promotes survival and evolution of NEPC cells. LSD1 is highly up-regulated in NEPC, and allosteric inhibitors targeting LSD1 can attenuate the viability of NEPC cells [143].

Apart from these, in the absence of RB1 and TP53, the placental gene PEG10 promotes cell cycle progression from G0/G1 and enhances the invasion of NEPC via transforming growth factor-β (TGF-β) signaling [144]. Targeting PEG10 with a specific short hairpin RNA (shRNA) can inhibit its positive activity in NEPC and considerably suppress tumor growth in patients with NEPC [111]. Both RB1 and TP53 make contributions to curbing lineage plasticity by inhibiting the expression of SOX2 [17] so as to suppress NEPC. Simultaneously, loss of RB1 facilitates lineage plasticity and metastasis of prostate adenocarcinoma initiated by PTEN mutation [123].

Liu et al. [145] discovered that prolonged ENZ treatment resulted in RB1 phosphorylation and transcriptional up-regulation of N-MYC, enhancing gene expression of NEPC. Fortunately, these activities can be suppressed by OLA or TALA. Furthermore, dinaciclib was found to inhibit RB1 phosphorylation, resulting in marked inhibition of NED when combined with OLA [145]. RB1 knockout enhances homeobox protein 5 (HOXB5) expression, and this effect can be further amplified by TP53 gene knockout. In addition, elevated HOXB5 expression promotes cell growth and invasion in PCa cell lines, indicating that HOXB5 partially accounts for the formation of NEPC [146]. TRIM59, a member of the tripartite motif (TRIM) protein family, exhibits a strong correlation with the development of NED and shorter patient survival in PCa following treatment with second-generation ARPIs. The expression of TRIM59 is up-regulated in NEPC that arises as a result of ARPI treatment. This up-regulation of TRIM59 in PCa drives NED by enhancing RB1 and TP53 degradation while also enhancing the activity of the transcription factor SOX2 [147]. Assuming that successful targeting of TRIM59 is achieved, reducing its expression level can mitigate its negative regulatory effects on RB1 and TP53 in NEPC, thereby inhibiting the occurrence of this aggressive subtype.

The anti-histamine drug ketotifen effectively induces apoptosis of NEPC tumor cells by targeting the interleukin-6 (IL-6)/signal transducer and activator of transcription 3 (STAT3) pathway, which reduces the survival rate of NEPC [68]. This effect resembles that of tumor suppressor genes and suggests its potential as a treatment for NEPC.

### Epigenetic regulatory factors and transcriptional factors

#### SRRM4 and REST

The RNA splicing factor serine/arginine repetitive matrix 4 (SRRM4) facilitates the NE transformation of PCa by inhibiting AR, which promotes the progression of NEPC by compromising the function of RB1 and TP53 [5]. In untreated PCa-adeno, low SRRM4 expression was detected in approximately 16% of tumor cores, and PCa-adeno treated with neoadjuvant hormone therapy for over 7 months had critically higher SRRM4 expression [79]. Therefore, the expression of SRRM4 in castration-resistant tumors is highly correlated with the emergence of NEPC and poor survival in patients. Fortunately, targeted inhibition of SRRM4 can effectively exert an anti-NEPC effect by inhibiting the aforementioned pathways. Yoshida et al. [148] extended the application of SRRM4 antisense oligonucleotide (ASO) medicine from SCLC to PCa cells exhibiting aberrant SRRM4 mRNA expression in vitro. They demonstrated that SRRM4 ASO successfully down-regulated SRRM4 expression and inhibited the viability of both SCLC and PCa cells in a dose-dependent manner. The data indicate that the gapmer oligonucleotide targeting SRRM4 (SRRM4 ASO) reduces cellular viability by restoring REST splicing, thereby exhibiting significant antitumor efficacy. Besides, small-molecule inhibitors (SMIs) may target the RNA-binding domain (RBD) of SRRM4 to block its recognition of RNA substrates, or directly inhibit the C-terminal domain critical for SRRM4’s splicing activity [149]. In addition, SRRM4 plays a crucial role in mediating the alternative splicing of REST, leading to the generation of a dominant negative form of REST [150].

REST is identified as one of the target genes regulated by SRRM4 and serves as a primary regulator in NED [5]. By inhibiting the expression of genes essential for NED, REST acts as a major negative regulator in neurogenesis [5]. However, cell cycle profile analysis conducted by Flores-Morales et al. [30] revealed that reducing REST levels in cell culture resulted in an increased number of cells in G1 phase and a decreased number of cells in S phase. Moreover, inhibition of REST may lead to the overexpression of neurogenes (N-MYC, AURKA, etc.) and promotion of the NE phenotype [43]. Multitudes of studies have demonstrated that loss of REST is a crucial factor affecting the origination of NEPC [30,151]. Additionally, REST plays a role as a negative feedback regulator on SRRM4 [30]. On this basis, Mishima et al. [152] investigated splice-switching oligonucleotides (SSOs) based on amido-bridged nucleic acids (AmNA) that target the splicing of REST as a novel therapeutic strategy. Their findings demonstrated that REST_SSO suppresses NE tumorigenesis by restoring functional REST in PCa xenograft models.

#### EZH2

EZH2, an epigenetic reprogramming factor, and its proteins exhibit remarkable activity in the majority of NEPC cases [20]. EZH2 appears to be a common mediator in signaling pathways triggered by all key molecular events in NEPC, including N-MYC amplification, cell cycle arrest, activated Wnt signaling, and loss of P53 or RB1 [153]. Luo et al. [154] discovered that EZH2 knockout in ENZ-induced NE phenotype C4-2 cells could substantially reduce the expression of NE markers. Meanwhile, RNA-seq and chromatin immunoprecipitation-seq (ChIP-seq) data along with preclinical studies using PDX support N-MYC’s induction of EZH2 transcriptional changes that alter the epigenetic programming of NEPC cells and promote their growth [20,154,155]. The combined effect of N-MYC and EZH2 promotes the expression of PCa NE phenotype as well as the development of AR resistance, migration of CRPC cells, and formation of NEPC [156]. Inhibiting EZH2 may potentially reverse N-MYC-induced suppression on genes [66], which could block ENZ and other drug-induced NED [154]. These findings suggest that targeting EZH2 could be a promising therapeutic strategy [111].

#### FOXA1 and FOXA2

The essential transcription factor FOXA1 exhibits decreased expression in NEPC [23]. One of the primary roles of FOXA1 is to act as a precursor factor for AR binding to chromatin and serve as a key regulator of AR’s transcriptional output [157,158]. FOXA1 is a downstream target of SRRM4 [138]. It can be reasonably inferred that inhibiting SRRM4 could alleviate the inhibition of FOXA1, thus playing a certain role in restraining the conversion of PCa to NEPC. Previously, FOXA1 was believed to function as both an oncogene and a tumor suppressor gene [23]. However, in NEPC, FOXA1 is reprogrammed as a NE-specific regulatory element with increased binding to regulatory elements rich in NEPC. It is surrounded by a super enhancer in NEPC and participates in the core regulation circuit of neuron pedigree transcription factor ASCL1 [158]. It also promotes cell proliferation in primary PCa [23]. Nevertheless, the majority of mainstream research findings indicate that FOXA1 functions as a negative regulator in the progression of PCa to NEPC, with its down-regulation observed in NEPC and its role in inhibiting NED [23,159]. In this context, we focus on FOXA1 as the central factor and present an overview of therapeutic effects achieved by targeting both its upstream and downstream pathways for NEPC treatment.

Another transcription factor, FOXA2, belonging to the same family as FOXA1, also plays a crucial role in NEPC. However, their roles are almost diametrically opposite. During the development of NEPC, there is a evident increase in DNA binding activity for FOXA2. Conversely, for FOXA1, not only does the number of binding sites decrease, but its overall DNA binding activity decreases as well [44,160]. The expression level of FOXA1 is negatively correlated with the expression level of characteristic genes for NEPC such as SOX2 [160]. The balance between FOXA1 and FOXA2 controls the transformation of PCa to NEPC [24]. As far as FOXA2 is concerned, it is considerably induced by ADT and coordinates the NE lineage during the transition from PCa to NEPC while driving the NED process. Due to its constant activation, NED always occurs, and even targeted knockdown might curb the progression of PCa toward an NEPC phenotype [24,160].

#### BRN2

The Pit-Oct-Unc (POU) domain transcription factor BRN2 serves as the principal regulator of NED and is directly transcribed by AR [41]. BRN2 is essential for the expression of NE terminal markers and invasive growth in CRPC induced by treatment targeting AR. Furthermore, it exhibits high expression and activation in NEPC and mCRPC [41]. It plays a pivotal role as one of the key regulators of NED in PCa [41]. MUC1-C resides upstream of BRN2 and induces an increase in BRN2 through MYC mediation, and silencing MUC1-C can down-regulate BRN2 [21]. These components constitute the unidirectional MUC1-C/MYC/BRN2/SOX2 pathway [21], in which targeted inhibition at any site can contribute to the treatment of NEPC. In addition, EVs selectively release BRN2 [42]. Encouragingly, inhibition using the GW4869 inhibitor resulted in reduced levels of EV-associated BRN2 [42], which may be a promising therapy for NEPC as well.

#### SOX2, ASCL1, and NEUROD1

CRPC facilitates the development and progression of NEPC by regulating transcriptional activity of SOX2 and inducing lineage differentiation of ASCL1 as well as NEUROD1 [25,109]. SOX2 plays a vital role in determining neural lineage-specific fate [51,161,162], and it is highly expressed in NEPC cells [25]. Database predictions and other experiments indicate that SOX2 may function as a transcription factor to initiate ASCL1 transcription, which induces NED [25].

The posttreatment NEPC can be classified into ASCL1 and NEUROD1 subtypes based on expression clusters. The gene expression and enrichment pathways differ between the 2 subtypes, as well as their invasive abilities and tissue characteristics. These differences in subtypes may impact the efficacy of drug treatment, suggesting the need for new therapeutic strategies for NEPC [109].

ASCL1 serves as a pivotal transcription factor in cellular NED [25]. It coordinates extensive molecular-level transcriptional reprogramming and plays an important part in driving neuronal cell differentiation and lineage specification [163]. Following ADT, the expression of prototypical neurogenic regulator ASCL1 is substantially up-regulated [25,163]. Above all, ASCL1 is indispensable for treatment-induced plasticity, and targeting ASCL1 weakens lineage plasticity and mitigates the emergence of fatal diseases [25].

In addition, NEUROD1, a neuron transcription factor, is independent of ASCL1 subtype and has similar epigenetic characteristics [109,110]. It binds to SOX2 at the same frequency in NEPC [109,110] and actively stimulates the growth of NEPC cells by forming a positive feedback loop with super enhancer [109,164]. Based on the heterogeneity of the 2 subtypes as targets, the study of new therapeutic strategies for NEPC may be a new direction of drug research.

#### OC2

The transcription factor OC2, a member of the ONECUT family, plays a crucial role in tumor growth and metastasis of PCa. OC2 exhibits a negative correlation with AR activity in PCa and is remarkably overexpressed in NEPC [32,165], thereby promoting the NED of PCa through regulation of hypoxia signals [32,166]. Additionally, REST directly inhibits OC2 as a central regulatory node in NEPC upstream [167], while the binding efficiency of REST’s upstream regulator HF1α to chromatin is regulated by OC2 [165]. OC2 also directly inhibits its downstream factor FOXA1 [32]. These findings suggest that targeting the REST/OC2/FOXA1 pathway may hold potential therapeutic value. Additionally, OC2 directly activates PEG10, which serves as a key regulator governing the transition of PCa to NEPC [167]. Recent reports have demonstrated that CSRM617 [167], an SMI of OC2, and TH-302 [168], a hypoxia-activated prodrug, effectively induce cell death in PCa cell lines with high expression levels of OC2 and obviously inhibit tumor growth [32]. In summary, targeting OC2 represents an attractive therapeutic approach for treating NEPC.

#### ZBTB7A

The transcription factor ZBTB7A, also known as Pokemon, contributes to promoting cell proliferation in NEPC [31]. In NEPC cell lines, ZBTB7A promotes G1/S conversion during the cell cycle and inhibits apoptosis. In addition, ZBTB7A counteracts negative regulation of the cell cycle by competitively inhibiting downstream sites and transcriptionally suppressing RB1 through its interaction with TP53 [31]. By silencing the tumorigenic function of ZBTB7A in NEPC cells, anti-NEPC effects can be achieved [31].

In the downstream region of ZBTB7A, RET, a receptor tyrosine kinase, exhibits critical enrichment in NEPC and demonstrates up-regulated gene expression [31,169]. This is an essential requirement for the proliferation of NEPC cells [169]. Silencing ZBTB7A inhibits cell proliferation in RET-dependent NEPC cells by regulating the cell cycle [31]. Additionally, the NEPC cell line NCI-H660 relies on RET expression for its proliferative capacity. Inhibitors targeting RET signal transduction pathways all possess certain anti-NEPC effects [31].

#### Others

In addition to the aforementioned targets, compounds, or drugs exhibiting potential targeted inhibition of NEPC, there is also potential in NEPC therapy in targeting dopamine receptor D2 (DRD2), apolipoprotein A-I (ApoA-I), nuclear receptor binding SET domain protein 2 (NSD2), and SWI/SNF complexes [170–173].

According to both bioinformatics and experimental evidence, ApoA-I, a crucial component of high-density lipoprotein (HDL), is found to be up-regulated in NEPC. This up-regulation, directly controlled by the MYC gene, contributes to increased survival, proliferation, invasion, and therapy resistance of NEPC [173]. From another perspective, targeted inhibition of ApoA-I, regulated by N-MYC, hinders the development of NEPC. DRD2 is found to be overexpressed in NEPC. However, a recent study has demonstrated that ONC-201, a DRD2 inhibitor targeting this receptor, effectively inhibits the development of CRPC into NEPC [172]. NSD2 catalyzes histone H3 lysine 36 demethylation, which plays an important role in the process of CRPC NED [171]. SWI/SNF complexes, also known as Brg/Brahma-associated factor (BAF) complexes, promote NED in PCa as well [170]. Targeted inhibition of these complexes may hold potential therapeutic implications in lineage plasticity and castration resistance, thereby contributing to anti-NEPC strategies [171,174,175]. These targets exhibit vital anti-NEPC value in clinical research on NEPC. More promisingly, an increasing number of clinical trials suggest the progress in treatment of NEPC in patients (Table 2). Taken together, it is certain that precise and effective treatment strategies will emerge soon.

**Table 2.** Clinical trials in men with NEPC

Study	Treatment arm(s)	Target	Phase	Status
NCT04702737	Tarlatamab (AMG 757)	DLL3	I	Completed
NCT02709889	Rovalpituzumab tesirine versus dexamethasone	DLL3	I/II	Terminated
NCT05652686	PT217	DLL3/ CD47	I	Recruiting
NCT05413421	ORIC-944	PRC2	I	Recruiting
NCT00973882	Carboplatin + Etoposide		II	Completed
NCT06062745	^18^F-fluciclovine + PET/CT		I	Recruiting
NCT05988918	ESK981	PIKfyve	II	Recruiting
NCT03910660	BXCL701 versus BXCL701 + Pembrolizumab	Dipeptidyl Peptidases	I/II	Active, not recruiting
NCT05691465	Lutetium Lu 177 dotatate	Somatostatin receptor	II	Recruiting
NCT01799278	MLN8237	AURKA	II	Completed
NCT01848067	Alisertib + Abiraterone acetate + Prednisone	AURKA	I/II	Completed
NCT02464007	rSIFN-co		I	Terminated
NCT02893917	OLA versus OLA + Cediranib	PARP	II	Active, not recruiting
NCT04592237	Cabazitaxel/carboplatin/cetrelimab (followed by niraparib) + Cetrelimab versus cabazitaxel/carboplatin/cetrelimab (followed by niraparib)		II	Active, not recruiting
NCT03902951	Abiraterone acetate/apalutamide/leuprolide acetate + Stereotactic body radiation therapy	AR	II	Active, not recruiting
NCT04926181	Apalutamide + Cetrelimab	AR	II	Terminated
NCT06094842	Bendamustine	Immune System	I	Withdrawn
NCT00423254	MKC1106-PP	Immune System	I	Completed
NCT01460134	CDX-1127	Immune System	I	Completed
NCT05000294	Atezolizumab + Tivozanib	Immune Checkpoint + VEGF-TKI	I/II	Recruiting
NCT03263650	Cabazitaxel/carboplatin/prednisone + OLA		II	Active, not recruiting
NCT05582031	Regorafenib + Tislelizumab		II	Withdrawn
NCT03649841	Radiation therapy versus radiation therapy + Antiandrogen therapy/abiraterone acetate/prednisone		II	Terminated
NCT05605522	[225]-FPI-2059 versus [111In]-FPI-2058		I	Active, not recruiting
NCT01794793	Pasireotide versus cabergoline	D2	IV	Completed
NCT03896503	Topotecan versus topotecan + Berzosertib (M6620)	Topoisomerase I, ATR	II	Active, not recruiting
NCT00379132	131-I-TM-601 (chlorotoxin)	HER2 EGFR	I	Completed
NCT01391143	MGA271	B7-H3	I	Completed
NCT00753415	V934 versus V935 hTERT vaccination		I	Completed
NCT00428220	Sunitinib malate	RTK		Completed
NCT01155258	Temsirolimus + Vinorelbine ditartrate		I	Completed
NCT01638533	Romidepsin	Histone Deacetylase	I	Completed
NCT00089362	Alvespimycin hydrochloride		I	Completed
NCT00031681	7-Hydroxystaurosporine + Irinotecan hydrochloride	Topoisomerase I	I	Completed
NCT00004074	Interleukin-12 + Trastuzumab	GPC3, HER2	I	Completed
NCT02860286	Tazemetostat	EZH2	II	Completed
NCT04179864	EPZ6438 (tazemetostat)	EZH2	Ib/II	Terminated
NCT03480646	CPI-1205 + Enzalutamide/abiraterone/prednisone	EZH2	Ib/II	Unknown
NCT03072238	Ipatasertib	PI3K/AKT/mTOR	III	Completed
NCT02881242	Trametinib (TMT212)	MEK/ERK	II	Active, not recruiting
NCT04631744	Cabozantinib	RET + KIT	II	Recruiting
NCT04446117	Cabozantinib + Atezolizumab	RET + KIT	III	Active, not recruiting
NCT05502315	Cabozantinib + Nivolumab	RET + KIT	II	Recruiting
NCT04471974	ZEN-3694	BET	II	Recruiting
NCT04986423	ZEN-3694	BET	IIb	Recruiting
NCT04628988	CC-90011	LSD1	I	Completed
NCT02998567	Guadecitabine (SGI-110)	DNMT	I	Recruiting
NCT05037500	Decitabine + Cedazuridine	DNMT	Ib	Terminated
NCT02125357	Abiraterone + Prednisone + enzalutamide		II	Completed
NCT02254785	Cabazitaxel + Abiraterone/enzalutamide	AR/Taxane Receptor	II	Unknown
NCT04015622	Enzalutamide + Docetaxel	AR/Taxane Receptor	II	Recruiting
NCT03473925	Navarixin (MK-7123) + Pembrolizumab (MK-3475)	CXCR2	II	Completed
NCT01695044	Abiraterone + Enzalutamide	PSMA	II	Completed
NCT00848718	MK-2206	ALK	I	Completed
NCT03333031	HS-196	HSP90 ATP Binding Domain	I	Terminated
NCT00546039	BONISTEIN		II	Unknown
NCT00513071	Saracatinib (AZD0530)	SRC	II	Completed
NCT03761017	Lorigerlimab	CTLA-4, PD-1	I	Completed
NCT03333616	Ipilimumab + Nivolumab	CTLA-4, PD-1	II	Active, not recruiting
NCT03866382	Ipilimumab + Cabozantinib + Nivolumab	CTLA-4, PD-1	II	Recruiting
NCT03629756	Etrumadenant (AB928) + Zimberelimab (AB122)	A2a/b Adenosine Receptor, PD-1	I	Completed
NCT03582475	Pembrolizumab + Etoposide/docetaxel/cisplatin/carboplatin	PD-1	I	Completed
NCT04802876	Spartalizumab + Tislelizumab	PD-1	II	Active, not recruiting
NCT00441337	MDX-1106	PD-1	I	Completed
NCT03365791	PDR001 + LAG525	PD1	II	Completed
NCT03517488	XmAb20717 (Vudalimab)	PD-1	I	Completed
NCT05585034	XmAb 808 + Keytruda (pembrolizumab)	PD-1	I	Active, not recruiting
NCT03849469	XmAb 22841 versus XmAb 22841 + Keytruda (pembrolizumab)	PD-1	I	Completed
NCT04848337	Pembrolizumab + Lenvatinib	PD-1	II	Active, not recruiting
NCT02834013	Nivolumab + Ipilimumab	PD-1	II	Active, not recruiting
NCT03036098	Nivolumab + Ipilimumab + Gemcitabine	PD-1	III	Active, not recruiting
NCT03774901	Avelumab	PD-1	I	Unknown
NCT03179410	Avelumab	PD-L1	II	Completed
NCT04709276	Nivolumab + Ipilimumab + Carboplatin + Cabazitaxel	PD-L1	II	Recruiting

AR, androgen receptor; DLL3, delta-like ligand 3; PD-1, programmed death 1; PD-L1, programmed cell death-ligand 1; DNMT, DNA methyltransferase; LSD1, lysine-specific demethylase 1; BET, bromodomain and extra-terminal; RET, rearranged during transfection proto-oncogene; MEK, mitogen-activated protein kinase; ERK, extracellular regulated protein kinases; RTK, small-molecule multitarget receptor tyrosine kinase; ALK, anaplastic lymphoma kinase; AURKA, Aurora kinase A; HSP90, heat shock protein 90; PSMA, prostate-specific membrane antigen; CXCR2, C-X-C motif chemokine receptor 2; HER2, human epidermal growth factor receptor 2; EGFR, epidermal growth factor receptor 2; B7-H3, a member of the B7 superfamily; SRC, a nonreceptor tyrosine kinase family; GPC3, glypican 3; D2, dopamine type receptor 2; ATR, ataxia telangiectasia and rad3-related kinase; CTLA-4, a member of immunoglobulin superfamily; VEGF-TKI, vascular endothelial growth factor–tyrosine kinase inhibitors

## TME in NEPC

The TME, an intricate network encompassing both the internal landscape and the external milieu surrounding tumoral cells, constitutes a sophisticated system that integrates blood vessels, immune cells, and the intricate tumor extracellular matrix. This dynamic environment transcends mere structural, functional, and metabolic facets of the tumor, profoundly influencing its progression and, in the context of PCa, its metastatic potential [176,177]. Moreover, extensive research has emphasized the pivotal significance of the TME in fostering the development of NEPC, a subtype characterized by unique biological and clinical features [178,179].

The cellular landscape of NEPC is dominated by several key players, including (a) prostatic epithelial cells, which serve as the primary neoplastic component; (b) tumor-associated macrophages (TAMs), pivotal in modulating immune responses and tumor progression; (c) myeloid-derived suppressor cells (MDSCs), which contribute to immune evasion and promote tumor growth; (d) cancer-associated fibroblasts (CAFs), critical modulators of the extracellular matrix and mediators of tumor–stromal interactions; (e) mesenchymal stem cells (MSCs), with their potential to differentiate into various stromal cell types and influence tumor behavior; and (f) vascular endothelial cells (VECs), fundamental for angiogenesis and the maintenance of the tumor’s vascular supply. Together, these cellular constituents orchestrate a complex interplay that shapes the progression and metastatic potential of NEPC.

### Prostatic epithelial cells

Prostatic epithelial cells are the most dominant cell subsets in the TME, occupying a paramount position within the TME, where they exert a pivotal role in modulating neighboring cellular components and indirectly fostering the emergence of NEPC.

The expression of forkhead box protein C2 (FOXC2) in prostate epithelial cells can directly promote the development of NEPC [180]. Additionally, these epithelial cells secrete factors such as bone morphogenetic protein 6 (BMP-6) and cyclooxygenase 2 (COX-2), which facilitate the phenotypic transition of TAMs from the antitumorigenic M1 subtype to the tumor-promoting M2 subtype [181].

### TAMs

TAMs are the predominant immune cell subset within the TME and exhibit 2 polarization states: the classically activated M1 subtype and the alternatively activated M2 subtype. M1 macrophages play a pivotal role in innate host defense and tumor cell eradication by generating reactive oxygen/nitrogen species (ROS/RNS) and pro-inflammatory cytokines such as IL-1β, IL-6, and tumor necrosis factor-α (TNF-α) [182]. In contrast, M2 macrophages secrete anti-inflammatory cytokines, including IL-10, IL-13, and TGF-β, which not only facilitate PCa progression but also may induce NED [182].

TAMs, as infiltrating macrophages within tumor tissue, have been implicated in promoting NED through the activation of downstream IL-6 signaling pathways [183].

### MDSCs

MDSCs, comprising a heterogeneous population of immature macrophages, dendritic cells, and granulocytes, exhibit potent immunosuppressive properties [184].

Under the influence of signaling molecules like STATs, MDSCs accumulate within the TME and secrete IL-6, which, in turn, activates STAT3 signaling to promote the progress of PCa [185]. At present, no studies have shown that MDSCs–IL-6–STAT3 can induce the formation of NEPC. However, MDSCs can indirectly promote the pathogenesis of NEPC by regulating other TME components. Specifically, MDSCs promote the differentiation of monocytes into M2-TAMs via paracrine IL-6 signaling while simultaneously inducing naïve T cells to differentiate into an IL-17-secreting T cell subset. The elevated IL-17 further stimulates PCa cells to up-regulate cyclooxygenase-2 (COX-2), which catalyzes the conversion of arachidonic acid into prostaglandin E2 (PGE2). PGE2 in turn reinforces monocyte differentiation into M2-TAMs, thereby activating downstream pathways that ultimately induce NED [186,187].

### CAFs

CAFs, the most abundant stromal cell type in the TME, play a crucial role in PCa progression, metastasis, and immune evasion [188]. Numerous studies have underscored the ability of CAFs to promote NEPC development through diverse signaling pathways, highlighting their pivotal role in tumor progression [188,189].

CAFs promote NEPC development through direct mechanisms or via epigenetic silencing of Ras protein activator-like 3 (RASAL3). Additionally, CD105-positive CAFs (CD105^+^ CAFs) induce NED by initiating a paracrine secreted frizzled-related protein 1 (SFRP1) signaling axis. Specifically, CD105^+^ CAFs activate this SFRP1-mediated paracrine pathway, leading to up-regulated expression of MYCN and AURKA, which collectively drive NEPC progression [189].

### MSCs

MSCs, a subset of pluripotent stem cells, have been shown to enhance the stem-like properties of PCa cells by promoting the secretion of chemokine-promoting factors like CCL5 [190]. This finding suggests that MSCs may also contribute to the promotion of NEPC. In an in vitro coculture system with PC3 cells (a human PCa cell line), Yu et al. [191] observed a time-dependent increase in TGF-β1 secretion within the conditioned medium. TGF-β1 activates downstream signaling pathways to induce NED. These findings collectively indicate that MSCs may promote NEPC pathogenesis.

### VECs

Angiogenesis, the formation of new blood vessels, is a hallmark of tumor progression. VECs play a central role in this process, with tumors promoting their proliferation to enhance angiogenesis within the TME. However, the specific correlation between VECs and NEPC formation remains an area of ongoing research and awaits further elucidation.

Currently, the exploration of the TME in the context of NEPC has emerged as a burgeoning research frontier. Elucidating the intricate interplay between NEPC and its TME holds immense promise for the development of targeted immunotherapies. Current research has begun to reveal the potential of immunotherapy for NEPC. For example, von Hardenberg et al. [192] have seminally reported the presence of PD-L1 molecular signatures in NEPC, underscoring the potential for immune checkpoint blockade in this subset. Supporting evidence comes from studies like that of Gu et al. [106], which demonstrated that combining platinum-based chemotherapy with PD-1 blockade evidently improved OS in patients with extensively metastatic NEPC. However, more comprehensive translational research is still needed to fully realize the clinical potential of this therapeutic approach. Notably, the TME in NEPC exhibits a distinct, relatively immune-deficient state compared to other primary and metastatic prostate adenocarcinomas [26]. While this presents therapeutic challenges, it also creates opportunities for developing novel immunotherapies. Recent advances show promising emerging strategies, such as the CEACAM5-targeted CAR-T cell therapy reported by Baek’s team [193], which demonstrated specific cytotoxicity against NEPC in preclinical studies. These findings collectively suggest that immunotherapy strategies specifically designed for NEPC’s unique TME characteristics may overcome its inherent resistance to conventional treatments and improve patient outcomes. The field of immunotherapy for advanced PCa is rapidly evolving, with continuous development and validation of new therapeutic approaches bringing renewed hope for NEPC patients.

## Conclusions and Future Perspectives

NEPC, an aggressive subtype of CRPC, is characterized by low or absent AR expression and loss of dependence on the AR pathway, representing the most malignant form of PCa currently known. It remains a key challenge in clinical practice. There is a better understanding on the activities and results of anti-androgen treatment on PCa because of advances of fundamental and translational biology, and several molecular changes that contribute to NEPC development have been found. In this field, 2 potential origin hypotheses have been proposed: de novo NEPC and treatment-induced NEPC. Current findings suggest that NEPC primarily arises in patients with CRPC following ARPI therapy. However, it is currently unknown how NE transdifferentiation specifically takes place and how emerging factors collaborate and contribute over time to the formation of treatment-induced NEPC.

Compared to primary PCa or CRPC, NEPC exhibits distinct clinical characteristics: it demonstrates a higher propensity for visceral metastases and is inherently insensitive to AR signaling. This AR-independent state leads to the natural resistance of NEPC to traditional ADT and second-generation AR pathway inhibitors. This therapeutic resistance not only stems from the inherent molecular heterogeneity of the tumor but also is achieved through a dynamic lineage plasticity mechanism—ADT-mediated inhibition of AR signaling activates NE transcriptional regulatory factors such as BRN2 and ASCL1, thereby promoting the transdifferentiation of tumor cells to an AR-independent state.

At present, the clinical benefits of the standard chemotherapy regimen based on cisplatin/etoposide are limited. This poor therapeutic effect stems from the multidrug resistance mechanism: (a) Epigenetic silencing of tumor suppressor genes (such as RB1) mediated by EZH2 promotes chemotherapy resistance, and (b) the tumor stem cell population maintains therapeutic resistance by continuously activating the Wnt/β-catenin pathway. These molecular characteristics highlight the urgency of developing novel therapeutic strategies targeting the unique biological properties of NEPC. More frustrating, with the limited methods of diagnosis, NEPC patients are typically diagnosed at an advanced stage, and once diagnosed, the survival time is limited to 1 to 2 years. This dire situation underscores the urgent need to develop more effective early diagnostic tools and targeted therapeutic strategies.

Fortunately, with research continuing to explore NEPC, important progress has been made in identifying genes and pathways that influence NEPC formation. In recent years, numerous studies have extensively investigated and unveiled potential avenues for targeted inhibition or activation of specific genes or pathways. These findings bring hopes for the future development of therapeutic drugs specifically designed to combat NEPC. As understanding of the factors influencing NEPC formation continues to deepen, targeted therapy is no longer a distant prospect but rather one of the most valuable treatment options.

Trop2, a cell surface glycoprotein, has been observed to undergo up-regulation in cells owing to the modulation of PARP1, thereby resulting in reduced DNA aggregation and histone methylation [27]. This pathway may be intricately linked to the emergence of NEPC. Trop2 thus holds promising potential as a novel target for inhibiting NEPC. Currently, MMU-132, an anti-TROP2 antibody–drug conjugate fused to an active topoisomerase inhibitor, has demonstrated efficacy against PCa [115]. However, clinical trials are yet to validate MMU-132’s ability to prevent the onset of NEPC.

Moreover, it is truly exciting that through combinatorial inhibition of different genes or pathways, progression of NEPC can be considerably impeded. A combined therapy utilizing PARP1 inhibitors and CD4/6 inhibitors targeting Trop2 has demonstrated greater efficacy than monotherapy [131]. Further clinical studies are required not only to determine the optimal treatment combinations for patients with NEPC but also to elucidate the necessity and sufficiency of Trop2 in NEPC development and clinically validate the efficacy of Trop2-targeting therapies. Additionally, inhibiting receptors on the surface of NEPC cells provides an alternative approach beyond targeting gene expression and signaling transduction pathways within cells. DLL3 is exclusively expressed on the surface of NEPC cells [120], rendering it a crucial target for the treatment of NEPC. Presently, molecular targeted therapies [120] and antibody–drug conjugates [26] targeting DLL3 are being rigorously developed. Moreover, NEPC cells have been observed to highly express other cell surface receptors, such as CEACAM5. CAR-T cell therapies targeting CEACAM5 have displayed promising therapeutic efficacy in NEPC cell lines [122], although in vitro experiments necessitate further refinement.

Furthermore, REST has emerged as a crucial regulator in suppressing the onset of NED, while SRRM4 can attenuate REST expression [5]. Currently, targeted drugs against SRRM4 are under investigation and are anticipated to emerge as a promising therapeutic approach for NEPC. Similarly, the loss of RB1, analogous to TP53, has been correlated with the transition from NED [22]. Other genes, including NK1R, CHRM4, and BRN2, have also been implicated in the transformation of mCRPC into NEPC. Nevertheless, given the intricate complexity of NEPC, epigenetic regulation and the modulation of the TME play pivotal roles. Therefore, comprehensive studies are warranted to further elucidate the underlying mechanisms of NEPC development.

In the future, we can also explore the relationship between NEPC and TME. Currently, research on TME is still restricted to the cellular level. An in-depth study of TME can not only elucidate the occurrence of NEPC but also offer a breakthrough for the immune cold environment of NEPC. Further integration of research into immunology and the TME holds the potential to usher in a promising future for immunotherapy in NEPC [194]. By delving deeper into the intricate interplay between the immune system and the unique TME of NEPC, we can unlock novel therapeutic avenues tailored to address the challenges posed by this aggressive malignancy. This collaborative approach, rooted in a profound understanding of both immunology and cancer biology, promises to revolutionize the treatment landscape for NEPC patients, offering hope for improved survival rates and quality of life.

Importantly, the heterogeneity of NEPC types offers the potential for more specific treatment options. NEPC can be classified into ASCL1 and NEUROD1 subtypes based on distinct expression patterns. The disparities in gene expression and enrichment pathways between these 2 subtypes influence the efficacy of drug therapy. For instance, AURKA, a target for SMIs like alisertib, has been found to be potentially more effective against the NEUROD1 subtype. In the future, the treatment of NEPC will increasingly consider the heterogeneity of its various subtypes. This approach will facilitate the development of more tailored and beneficial treatment strategies for patients. Undoubtedly, this represents a precious advancement in NEPC treatment.

Future attention should be primarily focused on strategies to prevent the development of NEPC, reduce the likelihood of PCa and CRPC transitioning into NEPC, and explore pharmacological interventions that can impede NEPC progression or reverse NED. By doing so, the fundamental resolution of NEPC formation can be achieved, which will lead to improved treatment options for both NEPC patients and those with PCa who have not yet undergone NE transformation.

## References

[1] Siegel RL, Giaquinto AN, Jemal A. Cancer statistics, 2024. CA Cancer J Clin. 2024;74(1):12–49. 38230766 10.3322/caac.21820

[2] Mottet N, van den Bergh RCN, Briers E, Van den Broeck T, Cumberbatch MG, De Santis M, Fanti S, Fossati N, Gandaglia G, Gillessen S, et al. EAU-EANM-ESTRO-ESUR-SIOG Guidelines on Prostate Cancer. Part II-2020 Update: Treatment of relapsing and metastatic prostate cancer. Eur Urol. 2021;79(2):263–282. 33039206 10.1016/j.eururo.2020.09.046

[3] Cai M, Song XL, Li XA, Chen M, Guo J, Yang DH, Chen Z, Zhao SC. Current therapy and drug resistance in metastatic castration-resistant prostate cancer. Drug Resist Updat. 2023;68:100962. 37068396 10.1016/j.drup.2023.100962

[4] Davies AH, Beltran H, Zoubeidi A. Cellular plasticity and the neuroendocrine phenotype in prostate cancer. Nat Rev Urol. 2018;15(5):271–286. 29460922 10.1038/nrurol.2018.22

[5] Li Y, Donmez N, Sahinalp C, Xie N, Wang Y, Xue H, Mo F, Beltran H, Gleave M, Wang Y, et al. SRRM4 drives neuroendocrine transdifferentiation of prostate adenocarcinoma under androgen receptor pathway inhibition. Eur Urol. 2017;71(1):68–78. 27180064 10.1016/j.eururo.2016.04.028

[6] Shiota M, Ushijima M, Tsukahara S, Nagakawa S, Blas L, Takamatsu D, Kobayashi S, Matsumoto T, Inokuchi J, Eto M. NR5A2/HSD3B1 pathway promotes cellular resistance to second-generation antiandrogen darolutamide. Drug Resist Updat. 2023;70:100990. 37478518 10.1016/j.drup.2023.100990

[7] Baek DS, Kim YJ, Vergara S, Conard A, Adams C, Calero G, Ishima R, Mellors JW, Dimitrov DS. A highly-specific fully-human antibody and CAR-T cells targeting CD66e/CEACAM5 are cytotoxic for CD66e-expressing cancer cells in vitro and in vivo. Cancer Lett. 2022;525:97–107. 34740610 10.1016/j.canlet.2021.10.041

[8] Yang C, Zhang J, Liao M, Yang Y, Wang Y, Yuan Y, Ouyang L. Folate-mediated one-carbon metabolism: A targeting strategy in cancer therapy. Drug Discov Today. 2021;26(3):817–825. 33316375 10.1016/j.drudis.2020.12.006

[9] Johnson RP, Ratnacaram CK, Kumar L, Jose J. Combinatorial approaches of nanotherapeutics for inflammatory pathway targeted therapy of prostate cancer. Drug Resist Updat. 2022;64:100865. 36099796 10.1016/j.drup.2022.100865

[10] Wang HT, Yao YH, Li BG, Tang Y, Chang JW, Zhang J. Neuroendocrine prostate cancer (NEPC) progressing from conventional prostatic adenocarcinoma: Factors associated with time to development of NEPC and survival from NEPC diagnosis-a systematic review and pooled analysis. J Clin Oncol. 2014;32(30):3383–3390. 25225419 10.1200/JCO.2013.54.3553

[11] Conteduca V, Oromendia C, Eng KW, Bareja R, Sigouros M, Molina A, Faltas BM, Sboner A, Mosquera JM, Elemento O, et al. Clinical features of neuroendocrine prostate cancer. Eur J Cancer. 2019;121:7–18. 31525487 10.1016/j.ejca.2019.08.011 PMC6803064

[12] Park JW, Lee JK, Sheu KM, Wang L, Balanis NG, Nguyen K, Smith BA, Cheng C, Tsai BL, Cheng D, et al. Reprogramming normal human epithelial tissues to a common, lethal neuroendocrine cancer lineage. Science. 2018;362(6410):91–95. 30287662 10.1126/science.aat5749 PMC6414229

[13] Lee JK, Phillips JW, Smith BA, Park JW, Stoyanova T, McCaffrey EF, Baertsch R, Sokolov A, Meyerowitz JG, Mathis C, et al. N-Myc drives neuroendocrine prostate cancer initiated from human prostate epithelial cells. Cancer Cell. 2016;29(4):536–547. 27050099 10.1016/j.ccell.2016.03.001 PMC4829466

[14] Lee DK, Liu Y, Liao L, Li W, Danielpour D, Xu J. Neuroendocrine prostate carcinoma cells originate from the p63-expressing basal cells but not the pre-existing adenocarcinoma cells in mice. Cell Res. 2019;29(5):420–422. 30778177 10.1038/s41422-019-0149-4 PMC6796947

[15] Quintanal-Villalonga Á, Chan JM, Yu HA, Pe’er D, Sawyers CL, Sen T, Rudin CM. Lineage plasticity in cancer: A shared pathway of therapeutic resistance. Nat Rev Clin Oncol. 2020;17(6):360–371. 32152485 10.1038/s41571-020-0340-z PMC7397755

[16] Bonkhoff H. Neuroendocrine differentiation in human prostate cancer. Morphogenesis, proliferation and androgen receptor status. Ann Oncol. 2001;12(Suppl 2):S141–S144. 11762342 10.1093/annonc/12.suppl_2.s141

[17] Liu S, Alabi BR, Yin Q, Stoyanova T. Molecular mechanisms underlying the development of neuroendocrine prostate cancer. Semin Cancer Biol. 2022;86(Pt 3):57–68. 35597438 10.1016/j.semcancer.2022.05.007

[18] Masone MC. A non-invasive approach for NEPC diagnosis. Nat Rev Urol. 2022;19(2):67. 10.1038/s41585-022-00566-5 35031785

[19] Ton AT, Singh K, Morin H, Ban F, Leblanc E, Lee J, Lallous N, Cherkasov A. Dual-inhibitors of N-Myc and AURKA as potential therapy for neuroendocrine prostate cancer. Int J Mol Sci. 2020;21(21):8277. 33167327 10.3390/ijms21218277 PMC7663809

[20] Dardenne E, Beltran H, Benelli M, Gayvert K, Berger A, Puca L, Cyrta J, Sboner A, Noorzad Z, MacDonald T, et al. N-Myc induces an EZH2-mediated transcriptional program driving neuroendocrine prostate cancer. Cancer Cell. 2016;30(4):563–577. 27728805 10.1016/j.ccell.2016.09.005 PMC5540451

[21] Yasumizu Y, Rajabi H, Jin C, Hata T, Pitroda S, Long MD, Hagiwara M, Li W, Hu Q, Liu S, et al. MUC1-C regulates lineage plasticity driving progression to neuroendocrine prostate cancer. Nat Commun. 2020;11(1):338. 31953400 10.1038/s41467-019-14219-6 PMC6969104

[22] Chen J, Shi M, Chuen Choi SY, Wang Y, Lin D, Zeng H, Wang Y. Genomic alterations in neuroendocrine prostate cancer: A systematic review and meta-analysis. BJUI Compass. 2023;4(3):256–265. 37025467 10.1002/bco2.212 PMC10071089

[23] Dong HY, Ding L, Zhou TR, Yan T, Li J, Liang C. FOXA1 in prostate cancer. Asian J Androl. 2023;25(3):287–295. 36018068 10.4103/aja202259 PMC10226509

[24] Han M, Li F, Zhang Y, Dai P, He J, Li Y, Zhu Y, Zheng J, Huang H, Bai F, et al. FOXA2 drives lineage plasticity and KIT pathway activation in neuroendocrine prostate cancer. Cancer Cell. 2022;40(11):1306–1323.e1308. 36332622 10.1016/j.ccell.2022.10.011

[25] Nie J, Zhang P, Liang C, Yu Y, Wang X. ASCL1-mediated ferroptosis resistance enhances the progress of castration-resistant prostate cancer to neurosecretory prostate cancer. Free Radic Biol Med. 2023;205:318–331. 37355053 10.1016/j.freeradbiomed.2023.06.006

[26] Mansfield AS, Hong DS, Hann CL, Farago AF, Beltran H, Waqar SN, Hendifar AE, Anthony LB, Taylor MH, Bryce AH, et al. A phase I/II study of rovalpituzumab tesirine in delta-like 3-expressing advanced solid tumors. NPJ Precis Oncol. 2021;5(1):74. 34354225 10.1038/s41698-021-00214-y PMC8342450

[27] Hsu EC, Rice MA, Bermudez A, Marques FJG, Aslan M, Liu S, Ghoochani A, Zhang CA, Chen YS, Zlitni A, et al. Trop2 is a driver of metastatic prostate cancer with neuroendocrine phenotype via PARP1. Proc Natl Acad Sci USA. 2020;117(4):2032–2042. 31932422 10.1073/pnas.1905384117 PMC6994991

[28] Wen YC, Tram VTN, Chen WH, Li CH, Yeh HL, Thuy Dung PV, Jiang KC, Li HR, Huang J, Hsiao M, et al. CHRM4/AKT/MYCN upregulates interferon alpha-17 in the tumor microenvironment to promote neuroendocrine differentiation of prostate cancer. Cell Death Dis. 2023;14(5):304. 37142586 10.1038/s41419-023-05836-7 PMC10160040

[29] Zhang XW, Li JY, Li L, Hu WQ, Tao Y, Gao WY, Ye ZN, Jia HY, Wang JN, Miao XK, et al. Neurokinin-1 receptor drives PKCa-AURKA/N-Myc signaling to facilitate the neuroendocrine progression of prostate cancer. Cell Death Dis. 2023;14(6):384. 37385990 10.1038/s41419-023-05894-x PMC10310825

[30] Flores-Morales A, Bergmann TB, Lavallee C, Batth TS, Lin D, Lerdrup M, Friis S, Bartels A, Kristensen G, Krzyzanowska A, et al. Proteogenomic characterization of patient-derived xenografts highlights the role of REST in neuroendocrine differentiation of castration-resistant prostate cancer. Clin Cancer Res. 2019;25(2):595–608. 30274982 10.1158/1078-0432.CCR-18-0729

[31] Bae SY, Bergom HE, Day A, Greene JT, Sychev ZE, Larson G, Corey E, Plymate SR, Freedman TS, Hwang JH, et al. ZBTB7A as a novel vulnerability in neuroendocrine prostate cancer. Front Endocrinol. 2023;14:1093332. 10.3389/fendo.2023.1093332 PMC10090553 37065756

[32] Choi WW, Boland JL, Lin J. ONECUT2 as a key mediator of androgen receptor-independent cell growth and neuroendocrine differentiation in castration-resistant prostate cancer. Cancer Drug Resist. 2022;5(1):165–170. 35582526 10.20517/cdr.2021.108 PMC8992592

[33] Alabi BR, Liu S, Stoyanova T. Current and emerging therapies for neuroendocrine prostate cancer. Pharmacol Ther. 2022;238:108255. 35905791 10.1016/j.pharmthera.2022.108255

[34] Gagnon R, Kish EK, Cook S, Takemura K, Cheng BYC, Bressler K, Heng DYC, Alimohamed N, Ruether D, Lee-Ying RM, et al. Real-world clinical outcomes and prognostic factors in neuroendocrine prostate cancer. Clin Genitourin Cancer. 2025;23:102274. 39689666 10.1016/j.clgc.2024.102274

[35] Gupta G, Lee CD, Guye ML, Van Sciver RE, Lee MP, Lafever AC, Pang A, Tang-Tan AM, Winston JS, Samli B, et al. Unmet clinical need: Developing prognostic biomarkers and precision medicine to forecast early tumor relapse, detect chemo-resistance and improve overall survival in high-risk breast cancer. Ann Breast Cancer Ther. 2020;4(1):48–57. 32542231 10.36959/739/525 PMC7295150

[36] Monaghan PJ, Robinson S, Rajdl D, Bossuyt PMM, Sandberg S, St John A, O'Kane M, Lennartz L, Röddiger R, Lord SJ, et al. Practical guide for identifying unmet clinical needs for biomarkers. EJIFCC. 2018;29(2):129–137. 30050396 PMC6053814

[37] Séguier D, Parent P, Duterque-Coquillaud M, Labreuche J, Fromont-Hankard G, Dariane C, Penel N, Villers A, Turpin A, Olivier J. Emergence of neuroendocrine tumors in patients treated with androgen receptor pathway inhibitors for metastatic prostate cancer: A systematic review and meta-analysis. Eur Urol Oncol. 2025;8(2):581–590. 39824723 10.1016/j.euo.2024.12.014

[38] Bernal A, Bechler AJ, Mohan K, Rizzino A, Mathew G. The current therapeutic landscape for metastatic prostate cancer. Pharmaceuticals. 2024;17(3):351. 38543137 10.3390/ph17030351 PMC10974045

[39] Bhinder B, Ferguson A, Sigouros M, Uppal M, Elsaeed AG, Bareja R, Alnajar H, Eng KW, Conteduca V, Sboner A, et al. Immunogenomic landscape of neuroendocrine prostate cancer. Clin Cancer Res. 2023;29(15):2933–2943. 37223924 10.1158/1078-0432.CCR-22-3743 PMC10524949

[40] Beltran H, Oromendia C, Danila DC, Montgomery B, Hoimes C, Szmulewitz RZ, Vaishampayan U, Armstrong AJ, Stein M, Pinski J, et al. A phase II trial of the Aurora kinase A inhibitor alisertib for patients with castration-resistant and neuroendocrine prostate cancer: Efficacy and biomarkers. Clin Cancer Res. 2019;25(1):43–51. 30232224 10.1158/1078-0432.CCR-18-1912 PMC6320304

[41] Bishop JL, Thaper D, Vahid S, Davies A, Ketola K, Kuruma H, Jama R, Nip KM, Angeles A, Johnson F, et al. The master neural transcription factor BRN2 is an androgen receptor-suppressed driver of neuroendocrine differentiation in prostate cancer. Cancer Discov. 2017;7(1):54–71. 27784708 10.1158/2159-8290.CD-15-1263

[42] Bhagirath D, Yang TL, Tabatabai ZL, Majid S, Dahiya R, Tanaka Y, Saini S. BRN4 is a novel driver of neuroendocrine differentiation in castration-resistant prostate cancer and is selectively released in extracellular vesicles with BRN2. Clin Cancer Res. 2019;25(21):6532–6545. 31371344 10.1158/1078-0432.CCR-19-0498 PMC6825556

[43] Chen WY, Thuy Dung PV, Yeh HL, Chen WH, Jiang KC, Li HR, Chen ZQ, Hsiao M, Huang J, Wen YC, et al. Targeting PKLR/MYCN/ROMO1 signaling suppresses neuroendocrine differentiation of castration-resistant prostate cancer. Redox Biol. 2023;62:102686. 36963289 10.1016/j.redox.2023.102686 PMC10060381

[44] Elhasasna H, Khan R, Bhanumathy KK, Vizeacoumar FS, Walke P, Bautista M, Dahiya DK, Maranda V, Patel H, Balagopal A, et al. A drug repurposing screen identifies fludarabine phosphate as a potential therapeutic agent for N-MYC overexpressing neuroendocrine prostate cancers. Cells. 2022;11(14):2246. 35883689 10.3390/cells11142246 PMC9317991

[45] Bhagirath D, Liston M, Patel N, Akoto T, Lui B, Yang TL, To DM, Majid S, Dahiya R, Tabatabai ZL, et al. MicroRNA determinants of neuroendocrine differentiation in metastatic castration-resistant prostate cancer. Oncogene. 2020;39(49):7209–7223. 33037409 10.1038/s41388-020-01493-8 PMC7718386

[46] Sandhu HS, Portman KL, Zhou X, Zhao J, Rialdi A, Sfakianos JP, Guccione E, Kyprianou N, Zhang B, Mulholland DJ. Dynamic plasticity of prostate cancer intermediate cells during androgen receptor-targeted therapy. Cell Rep. 2022;40(4):111123. 35905714 10.1016/j.celrep.2022.111123

[47] Ge R, Wang Z, Montironi R, Jiang Z, Cheng M, Santoni M, Huang K, Massari F, Lu X, Cimadamore A, et al. Epigenetic modulations and lineage plasticity in advanced prostate cancer. *Ann Oncol*. 2020;31(4):470–479. 10.1016/j.annonc.2020.02.002 32139297

[48] Bonkhoff H. Factors implicated in radiation therapy failure and radiosensitization of prostate cancer. Prostate Cancer. 2012;2012:593241. 22229096 10.1155/2012/593241 PMC3200271

[49] Hvamstad T, Jordal A, Hekmat N, Paus E, Fosså SD. Neuroendocrine serum tumour markers in hormone-resistant prostate cancer. Eur Urol. 2003;44:215–221. 12875941 10.1016/s0302-2838(03)00257-4

[50] Abbott T, Ng K, Nobes J, Muehlschlegel P. Small-cell carcinoma of the prostate - challenges of diagnosis and treatment: A next of kin and physician perspective piece. Oncol Ther. 2023;11(3):291–301. 37358792 10.1007/s40487-023-00238-3 PMC10447819

[51] Kwon OJ, Zhang L, Jia D, Zhou Z, Li Z, Haffner M, Lee JK, True L, Morrissey C, Xin L. De novo induction of lineage plasticity from human prostate luminal epithelial cells by activated AKT1 and c-Myc. Oncogene. 2020;39(48):7142–7151. 33009488 10.1038/s41388-020-01487-6 PMC7704645

[52] Aggarwal R, Huang J, Alumkal JJ, Zhang L, Feng FY, Thomas GV, Weinstein AS, Friedl V, Zhang C, Witte ON, et al. Clinical and genomic characterization of treatment-emergent Small-cell neuroendocrine prostate cancer: A multi-institutional prospective study. J Clin Oncol. 2018;36(24):2492–2503. 29985747 10.1200/JCO.2017.77.6880 PMC6366813

[53] Wishahi M. Treatment-induced neuroendocrine prostate cancer and de novo neuroendocrine prostate cancer: Identification, prognosis and survival, genetic and epigenetic factors. World J Clin Cases. 2024;12:2143–2146. 38808339 10.12998/wjcc.v12.i13.2143 PMC11129135

[54] Aggarwal R, Zhang T, Small EJ, Armstrong AJ. Neuroendocrine prostate cancer: Subtypes, biology, and clinical outcomes. J Natl Compr Cancer Netw. 2014;12(12):719–726. 10.6004/jnccn.2014.0073 24812138

[55] Wang Y, Wang Y, Ci X, Choi SYC, Crea F, Lin D, Wang Y. Molecular events in neuroendocrine prostate cancer development. Nat Rev Urol. 2021;18(10):581–596. 34290447 10.1038/s41585-021-00490-0 PMC10802813

[56] Beltran H, Rickman DS, Park K, Chae SS, Sboner A, MacDonald TY, Wang Y, Sheikh KL, Terry S, Tagawa ST, et al. Molecular characterization of neuroendocrine prostate cancer and identification of new drug targets. Cancer Discov. 2011;1(6):487–495. 22389870 10.1158/2159-8290.CD-11-0130 PMC3290518

[57] Yamada Y, Beltran H. Clinical and biological features of neuroendocrine prostate cancer. Curr Oncol Rep. 2021;23(2):15. 33433737 10.1007/s11912-020-01003-9 PMC7990389

[58] Sreekumar A, Saini S. Role of transcription factors and chromatin modifiers in driving lineage reprogramming in treatment-induced neuroendocrine prostate cancer. Front Cell Dev Biol. 2023;11:1075707. 36711033 10.3389/fcell.2023.1075707 PMC9879360

[59] Davies A, Zoubeidi A, Selth LA. The epigenetic and transcriptional landscape of neuroendocrine prostate cancer. Endocr Relat Cancer. 2020;27(2):R35–R50. 31804971 10.1530/ERC-19-0420

[60] Iwamoto H, Nakagawa R, Makino T, Kadomoto S, Yaegashi H, Nohara T, Shigehara K, Izumi K, Kadono Y, Mizokami A. Treatment outcomes in neuroendocrine prostate cancer. Anticancer Res. 2022;42(4):2167–2176. 35347041 10.21873/anticanres.15699

[61] Heimdorfer D, Artamonova N, Culig Z, Heidegger I. Unraveling molecular characteristics and tumor microenvironment dynamics of neuroendocrine prostate cancer. J Cancer Res Clin Oncol. 2024;150(10):462. 39412660 10.1007/s00432-024-05983-0 PMC11485041

[62] Tritschler S, Erdelkamp R, Stief C, Hentrich M. Neuroendocrine prostate cancer. Pathologe. 2018;39:333–343. 10.1007/s00292-018-0453-7 29946852

[63] Aparicio AM, Harzstark AL, Corn PG, Wen S, Araujo JC, Tu SM, Pagliaro LC, Kim J, Millikan RE, Ryan C, et al. Platinum-based chemotherapy for variant castrate-resistant prostate cancer. Clin Cancer Res. 2013;19(13):3621–3630. 23649003 10.1158/1078-0432.CCR-12-3791 PMC3699964

[64] Beltran H, Hruszkewycz A, Scher HI, Hildesheim J, Isaacs J, Yu EY, Kelly K, Lin D, Dicker A, Arnold J, et al. The role of lineage plasticity in prostate cancer therapy resistance. Clin Cancer Res. 2019;25(23):6916–6924. 31363002 10.1158/1078-0432.CCR-19-1423 PMC6891154

[65] Zamora I, Freeman MR, Encío IJ, Rotinen M. Targeting key players of neuroendocrine differentiation in prostate cancer. Int J Mol Sci. 2023;24(18):13673. 37761978 10.3390/ijms241813673 PMC10531052

[66] Berger A, Brady NJ, Bareja R, Robinson B, Conteduca V, Augello MA, Puca L, Ahmed A, Dardenne E, Lu X, et al. N-Myc-mediated epigenetic reprogramming drives lineage plasticity in advanced prostate cancer. J Clin Invest. 2019;129(9):3924–3940. 31260412 10.1172/JCI127961 PMC6715370

[67] Beltran H, Tagawa ST, Park K, MacDonald T, Milowsky MI, Mosquera JM, Rubin MA, Nanus DM. Challenges in recognizing treatment-related neuroendocrine prostate cancer. J Clin Oncol. 2012;30(36):e386–e389. 23169519 10.1200/JCO.2011.41.5166

[68] Ji Y, Liu B, Chen L, Li A, Shen K, Su R, Zhang W, Zhu Y, Wang Q, Xue W. Repurposing ketotifen as a therapeutic strategy for neuroendocrine prostate cancer by targeting the IL-6/STAT3 pathway. Cell Oncol. 2023;46(5):1445–1456. 10.1007/s13402-023-00822-9 PMC12974647 37120492

[69] Teng M, Guo J, Xu X, Ci X, Mo Y, Kohen Y, Ni Z, Chen S, Guo WY, Bakht M, et al. Circular RMST cooperates with lineage-driving transcription factors to govern neuroendocrine transdifferentiation. Cancer Cell. 2025;43(5):891–904.e10. 40250444 10.1016/j.ccell.2025.03.027 PMC12991876

[70] Haffner MC, Morris MJ, Ding CKC, Sayar E, Mehra R, Robinson B, True LD, Gleave M, Lotan TL, Aggarwal R, et al. Framework for the pathology workup of metastatic castration-resistant prostate cancer biopsies. Clin Cancer Res. 2025;31(3):466–478. 39589343 10.1158/1078-0432.CCR-24-2061 PMC11790385

[71] Korsen JA, Gutierrez JA, Tully KM, Carter LM, Samuels ZV, Khitrov S, Poirier JT, Rudin CM, Chen Y, Morris MJ, et al. Delta-like ligand 3-targeted radioimmunotherapy for neuroendocrine prostate cancer. Proc Natl Acad Sci USA. 2022;119(27): Article e2203820119. 35759660 10.1073/pnas.2203820119 PMC9271187

[72] Hansson J, Abrahamsson PA. Neuroendocrine pathogenesis in adenocarcinoma of the prostate. Ann Oncol. 2001;12(Suppl 2):S145–S152. 11762343 10.1093/annonc/12.suppl_2.s145

[73] Cheng L, Yang T, Zhang J, Gao F, Yang L, Tao W. The application of radiolabeled targeted molecular probes for the diagnosis and treatment of prostate cancer. Korean J Radiol. 2023;24(6):574–589. 37271211 10.3348/kjr.2022.1002 PMC10248352

[74] Labrecque MP, Brown LG, Coleman IM, Lakely B, Brady NJ, Lee JK, Nguyen HM, Li D, Hanratty B, Haffner MC, et al. RNA splicing factors SRRM3 and SRRM4 distinguish molecular phenotypes of castration-resistant neuroendocrine prostate cancer. Cancer Res. 2021;81:4736–4750. 34312180 10.1158/0008-5472.CAN-21-0307 PMC8448969

[75] Labrecque MP, Coleman IM, Brown LG, True LD, Kollath L, Lakely B, Nguyen HM, Yang YC, da Costa RMG, Kaipainen A, et al. Molecular profiling stratifies diverse phenotypes of treatment-refractory metastatic castration-resistant prostate cancer. J Clin Invest. 2019;129(10):4492–4505. 31361600 10.1172/JCI128212 PMC6763249

[76] Epstein JI, Amin MB, Beltran H, Lotan TL, Mosquera JM, Reuter VE, Robinson BD, Troncoso P, Rubin MA. Proposed morphologic classification of prostate cancer with neuroendocrine differentiation. Am J Surg Pathol. 2014;38(6):756–767. 24705311 10.1097/PAS.0000000000000208 PMC4112087

[77] Fine SW. Neuroendocrine tumors of the prostate. Mod Pathol. 2018;31(S1):S122–S132. 29297494 10.1038/modpathol.2017.164

[78] Kemble J, Kwon ED, Karnes RJ. Addressing the need for more therapeutic options in neuroendocrine prostate cancer. Expert Rev Anticancer Ther. 2023;23(2):177–185. 36698089 10.1080/14737140.2023.2173174

[79] Li Y, Zhang Q, Lovnicki J, Chen R, Fazli L, Wang Y, Gleave M, Huang J, Dong X. SRRM4 gene expression correlates with neuroendocrine prostate cancer. Prostate. 2019;79(1):96–104. 30155992 10.1002/pros.23715

[80] Wang W, Epstein JI. Small cell carcinoma of the prostate. A morphologic and immunohistochemical study of 95 cases. Am J Surg Pathol. 2008;32(1):65–71. 18162772 10.1097/PAS.0b013e318058a96b

[81] Taher A, Jensen CT, Yedururi S, Surasi DS, Faria SC, Bathala TK, Mujtaba B, Bhosale P, Wagner-Bartak N, Morani AC. Imaging of neuroendocrine prostatic carcinoma. Cancers (Basel). 2021;13(22):5765. 34830919 10.3390/cancers13225765 PMC8616225

[82] Lee CF, Chen YA, Hernandez E, Pong RC, Ma S, Hofstad M, Kapur P, Zhau H, Chung LWK, Lai CH, et al. The central role of sphingosine kinase 1 in the development of neuroendocrine prostate cancer (NEPC): A new targeted therapy of NEPC. Clin Transl Med. 2022;12(2): Article e695. 35184376 10.1002/ctm2.695 PMC8858611

[83] Belge Bilgin G, Lucien-Matteoni F, Chaudhuri AA, Orme JJ, Childs DS, Muniz M, Li GG, Chauhan PS, Lee SB, Gupta S, et al. Current and future directions in theranostics for neuroendocrine prostate cancer. Cancer Treat Rev. 2025;136:102941. 40239461 10.1016/j.ctrv.2025.102941

[84] Dondi F, Antonelli A, Suardi N, Guerini AE, Albano D, Lucchini S, Camoni L, Treglia G, Bertagna F. PET/CT and conventional imaging for the assessment of neuroendocrine prostate cancer: A systematic review. Cancers. 2023;15(17):4404. 37686680 10.3390/cancers15174404 PMC10486674

[85] Kim J, Lee S, Kim D, Kim HJ, Oh KT, Kim SJ, Choi YD, Giesel FL, Kopka K, Hoepping A, et al. Combination of [^18^F]FDG and [^18^F]PSMA-1007 PET/CT predicts tumour aggressiveness at staging and biochemical failure postoperatively in patients with prostate cancer. Eur J Nucl Med Mol Imaging. 2024;51(6):1763–1772. 38200396 10.1007/s00259-023-06585-7

[86] Shen K, Liu B, Zhou X, Ji Y, Chen L, Wang Q, Xue W. The evolving role of ^18^F-FDG PET/CT in diagnosis and prognosis prediction in progressive prostate cancer. Front Oncol. 2021;11:683793. 34395251 10.3389/fonc.2021.683793 PMC8358601

[87] Spratt DE, Gavane S, Tarlinton L, Fareedy SB, Doran MG, Zelefsky MJ, Osborne JR. Utility of FDG-PET in clinical neuroendocrine prostate cancer. Prostate. 2014;74(11):1153–1159. 24913988 10.1002/pros.22831 PMC4355960

[88] Jin W, Yan L, Li L, Luo Y, Qiao J, Peng Q, Zhu Z, Zhu L, Kung HF. PSMA and SSTR2 dual-targeting theranostic agents for neuroendocrine-differentiated prostate cancer (NEPC). J Med Chem. 2025;68(2):1984–1993. 39791476 10.1021/acs.jmedchem.4c02768

[89] Mori H, Nakajima K, Kadomoto S, Mizokami A, Ikeda H, Wakabayashi H, Kinuya S. Imaging somatostatin receptor activity in neuroendocrine-differentiated prostate cancer. Intern Med. 2018;57(21):3123–3128. 29877274 10.2169/internalmedicine.0630-17 PMC6262717

[90] Chandekar KR, Prashanth A, Vinjamuri S, Kumar R. FAPI PET/CT imaging-an updated review. Diagnostics. 2023;13(12):2018. 37370912 10.3390/diagnostics13122018 PMC10297281

[91] Ge Q, Zhang ZY, Li SN, Ma JQ, Zhao Z. Liquid biopsy: Comprehensive overview of circulating tumor DNA (Review). Oncol Lett. 2024;28(5):548. 39319213 10.3892/ol.2024.14681 PMC11420644

[92] Borea R, Saldanha EF, Maheswaran S, Nicolo E, Singhal S, Pontolillo L, de Miguel Perez D, Venetis K, Dipasquale A, Ghazali N, et al. Cancer in a drop: Advances in liquid biopsy in 2024. Crit Rev Oncol Hematol. 2025;213:104776. 40447209 10.1016/j.critrevonc.2025.104776

[93] Beltran H, Romanel A, Conteduca V, Casiraghi N, Sigouros M, Franceschini GM, Orlando F, Fedrizzi T, Ku SY, Dann E, et al. Circulating tumor DNA profile recognizes transformation to castration-resistant neuroendocrine prostate cancer. J Clin Invest. 2020;130(4):1653–1668. 32091413 10.1172/JCI131041 PMC7108892

[94] Fonseca NM, Maurice-Dror C, Herberts C, Tu W, Fan W, Murtha AJ, Kollmannsberger C, Kwan EM, Parekh K, Schönlau E, et al. Prediction of plasma ctDNA fraction and prognostic implications of liquid biopsy in advanced prostate cancer. Nat Commun. 2024;15(1):1828. 38418825 10.1038/s41467-024-45475-w PMC10902374

[95] Menyailo ME, Tretyakova MS, Denisov EV. Heterogeneity of circulating tumor cells in breast cancer: Identifying metastatic seeds. Int J Mol Sci. 2020;21(5):1696. 32121639 10.3390/ijms21051696 PMC7084665

[96] Lin D, Shen L, Luo M, Zhang K, Li J, Yang Q, Zhu F, Zhou D, Zheng S, Chen Y, et al. Circulating tumor cells: Biology and clinical significance. Signal Transduct Target Ther. 2021;6(1):404. 34803167 10.1038/s41392-021-00817-8 PMC8606574

[97] Liu Q, Zhang H, Jiang X, Qian C, Liu Z, Luo D. Factors involved in cancer metastasis: A better understanding to “seed and soil” hypothesis. Mol Cancer. 2017;16(1):176. 29197379 10.1186/s12943-017-0742-4 PMC5712107

[98] Wang X, Wang L, Lin H, Zhu Y, Huang D, Lai M, Xi X, Huang J, Zhang W, Zhong T. Research progress of CTC, ctDNA, and EVs in cancer liquid biopsy. Front Oncol. 2024;14:1303335. 38333685 10.3389/fonc.2024.1303335 PMC10850354

[99] Beltran H, Jendrisak A, Landers M, Mosquera JM, Kossai M, Louw J, Krupa R, Graf RP, Schreiber NA, Nanus DM, et al. The initial detection and partial characterization of circulating tumor cells in neuroendocrine prostate cancer. Clin Cancer Res. 2016;22(6):1510–1519. 26671992 10.1158/1078-0432.CCR-15-0137 PMC4990782

[100] DeLucia DC, Cardillo TM, Ang L, Labrecque MP, Zhang A, Hopkins JE, de Sarkar N, Coleman I, da Costa RMG, Corey E, et al. Regulation of CEACAM5 and therapeutic efficacy of an anti-CEACAM5-SN38 antibody-drug conjugate in neuroendocrine prostate cancer. Clin Cancer Res. 2021;27(3):759–774. 33199493 10.1158/1078-0432.CCR-20-3396 PMC7854497

[101] Korsen JA, Kalidindi TM, Khitrov S, Samuels ZV, Chakraborty G, Gutierrez JA, Poirier JT, Rudin CM, Chen Y, Morris MJ, et al. Molecular imaging of neuroendocrine prostate cancer by targeting delta-like ligand 3. J Nucl Med. 2022;63(9):1401–1407. 35058323 10.2967/jnumed.121.263221 PMC9454466

[102] Shen M, Liu S, Stoyanova T. The role of Trop2 in prostate cancer: An oncogene, biomarker, and therapeutic target. Am J Clin Exp Urol. 2021;9(1):73–87. 33816696 PMC8012837

[103] Sperger JM, Helzer KT, Stahlfeld CN, Jiang D, Singh A, Kaufmann KR, Niles DJ, Heninger E, Rydzewski NR, Wang L, et al. Expression and therapeutic targeting of TROP-2 in treatment-resistant prostate cancer. Clin Cancer Res. 2023;29(12):2324–2335. 36939530 10.1158/1078-0432.CCR-22-1305 PMC10261916

[104] Liu S, Hawley SJ, Kunder CA, Hsu EC, Shen M, Westphalen L, Auman H, Newcomb LF, Lin DW, Nelson PS, et al. High expression of Trop2 is associated with aggressive localized prostate cancer and is a candidate urinary biomarker. Sci Rep. 2024;14(1):486. 38177207 10.1038/s41598-023-50215-z PMC10766957

[105] Huang W, Zhang Y, Cao M, Wu Y, Jiao F, Chu Z, Zhou X, Li L, Xu D, Pan X, et al. ImmunoPET imaging of Trop2 in patients with solid tumours. EMBO Mol Med. 2024;16(5):1143–1161. 38565806 10.1038/s44321-024-00059-5 PMC11099157

[106] Gu Y, Ly A, Rodriguez S, Zhang H, Kim J, Mao Z, Sachdeva A, Zomorodian N, Pellegrini M, Li G, et al. PD-1 blockade plus cisplatin-based chemotherapy in patients with small cell/neuroendocrine bladder and prostate cancers - PubMed. Cell Rep Med. 2024;5(11):101824. 39536751 10.1016/j.xcrm.2024.101824 PMC11604497

[107] Wang L-Y, Hung C-L, Wang T-C, Hsu H-C, Kung H-J, Lin K-H. PROTACs as therapeutic modalities for drug discovery in castration-resistant prostate cancer. Annu Rev Pharmacol Toxicol. 2025;65(1):375–376. 39116434 10.1146/annurev-pharmtox-030624-110238

[108] Guo Y, Cheng R, Wang Y, Gonzalez ME, Zhang H, Liu Y, Kleer CG, Xue L. Regulation of EZH2 protein stability: New mechanisms, roles in tumorigenesis, and roads to the clinic. EBioMedicine. 2024;100:104972. 38244292 10.1016/j.ebiom.2024.104972 PMC10835131

[109] Cejas P, Xie Y, Font-Tello A, Lim K, Syamala S, Qiu X, Tewari AK, Shah N, Nguyen HM, Patel RA, et al. Subtype heterogeneity and epigenetic convergence in neuroendocrine prostate cancer. Nat Commun. 2021;12(1):5775. 34599169 10.1038/s41467-021-26042-z PMC8486778

[110] Wang Z, Liu C, Zheng S, Yao Y, Wang S, Wang X, Yin E, Zeng Q, Zhang C, Zhang G, et al. Molecular subtypes of neuroendocrine carcinomas: A cross-tissue classification framework based on five transcriptional regulators. Cancer Cell. 2024;42(6):1106–1125. 38788718 10.1016/j.ccell.2024.05.002

[111] Okasho K, Mizuno K, Fukui T, Lin YY, Kamiyama Y, Sunada T, Li X, Kimura H, Sumiyoshi T, Goto T, et al. Establishment and characterization of a novel treatment-related neuroendocrine prostate cancer cell line KUCaP13. Cancer Sci. 2021;112:2781–2791. 33960594 10.1111/cas.14935 PMC8253279

[112] True LD, Buhler K, Quinn J, Williams E, Nelson PS, Clegg N, Macoska JA, Norwood T, Liu A, Ellis W, et al. A neuroendocrine/small cell prostate carcinoma xenograft—LuCaP 49. Am J Pathol. 2002;161(2):705–715. 12163395 10.1016/S0002-9440(10)64226-5 PMC1850754

[113] Risbridger GP, Clark AK, Porter LH, Toivanen R, Bakshi A, Lister NL, Pook D, Pezaro CJ, Sandhu S, Keerthikumar S, et al. The MURAL collection of prostate cancer patient-derived xenografts enables discovery through preclinical models of uro-oncology. Nat Commun. 2021;12(1):5049. 34413304 10.1038/s41467-021-25175-5 PMC8376965

[114] Ajkunic A, Sayar E, Roudier MP, Patel RA, Coleman IM, de Sarkar N, Hanratty B, Adil M, Zhao J, Zaidi S, et al. Assessment of cell surface targets in metastatic prostate cancer: Expression landscape and molecular correlates. Res Sq. 2023. 10.1038/s41698-024-00599-6 PMC11101486 38760413

[115] Stoyanova T, Goldstein AS, Cai H, Drake JM, Huang J, Witte ON. Regulated proteolysis of Trop2 drives epithelial hyperplasia and stem cell self-renewal via beta-catenin signaling. Genes Dev. 2012;26(20):2271–2285. 23070813 10.1101/gad.196451.112 PMC3475800

[116] Zhang XW, Li JY, Li L, Hu WQ, Tao Y, Gao WY, Ye ZN, Jia HY, Wang JN, Miao XK, et al. Neurokinin-1 receptor drives PKCɑ-AURKA/N-Myc signaling to facilitate the neuroendocrine progression of prostate cancer. Cell Death Dis. 2023;14(6):384. 37385990 10.1038/s41419-023-05894-x PMC10310825

[117] Reina-Campos M, Linares JF, Duran A, Cordes T, L'Hermitte A, Badur MG, Bhangoo MS, Thorson PK, Richards A, Rooslid T, et al. Increased serine and one-carbon pathway metabolism by PKClambda/iota deficiency promotes neuroendocrine prostate cancer. Cancer Cell. 2019;35(3):385–400.e389. 30827887 10.1016/j.ccell.2019.01.018 PMC6424636

[118] Chen WY, Wen YC, Lin SR, Yeh HL, Jiang KC, Chen WH, Lin YS, Zhang Q, Liew PL, Hsiao M, et al. Nerve growth factor interacts with CHRM4 and promotes neuroendocrine differentiation of prostate cancer and castration resistance. Commun Biol. 2021;4(1):22. 33398073 10.1038/s42003-020-01549-1 PMC7782543

[119] Chen X, Yin L, Xu H, Rong J, Feng M, Jiang D, Bai Y. Knockdown of RhoA expression reverts enzalutamide resistance via the p38 MAPK pathway in castration-resistant prostate cancer. Recent Pat Anticancer Drug Discov. 2023;18(1):92–99. 35339190 10.2174/1574892817666220325151555

[120] Puca L, Gavyert K, Sailer V, Conteduca V, Dardenne E, Sigouros M, Isse K, Kearney M, Vosoughi A, Fernandez L, et al. Delta-like protein 3 expression and therapeutic targeting in neuroendocrine prostate cancer. Sci Transl Med. 2019;11(484): Article eaav0891. 30894499 10.1126/scitranslmed.aav0891 PMC6525633

[121] Giffin MJ, Cooke K, Lobenhofer EK, Estrada J, Zhan J, Deegen P, Thomas M, Murawsky CM, Werner J, Liu S, et al. AMG 757, a half-life extended, DLL3-targeted bispecific T-cell engager, shows high potency and sensitivity in preclinical models of small-cell lung cancer. Clin Cancer Res. 2021;27(5):1526–1537. 33203642 10.1158/1078-0432.CCR-20-2845

[122] Lee JK, Bangayan NJ, Chai T, Smith BA, Pariva TE, Yun S, Vashisht A, Zhang Q, Park JW, Corey E, et al. Systemic surfaceome profiling identifies target antigens for immune-based therapy in subtypes of advanced prostate cancer. Proc Natl Acad Sci USA. 2018;115(19):E4473–E4482. 29686080 10.1073/pnas.1802354115 PMC5949005

[123] Ku SY, Rosario S, Wang Y, Mu P, Seshadri M, Goodrich ZW, Goodrich MM, Labbé DP, Gomez EC, Wang J, et al. Rb1 and Trp53 cooperate to suppress prostate cancer lineage plasticity, metastasis, and antiandrogen resistance. Science. 2017;355(6320):78–83. 28059767 10.1126/science.aah4199 PMC5367887

[124] Rodarte KE, Nir Heyman S, Guo L, Flores L, Savage TK, Villarreal J, Deng S, Xu L, Shah RB, Oliver TG, et al. Neuroendocrine differentiation in prostate cancer requires ASCL1. Cancer Res. 2024;84(21):3522–3537. 39264686 10.1158/0008-5472.CAN-24-1388 PMC11534540

[125] Unno K, Chalmers ZR, Pamarthy S, Vatapalli R, Rodriguez Y, Lysy B, Mok H, Sagar V, Han H, Yoo YA, et al. Activated ALK cooperates with N-Myc via Wnt/beta-catenin signaling to induce neuroendocrine prostate cancer. Cancer Res. 2021;81(8):2157–2170. 33637566 10.1158/0008-5472.CAN-20-3351 PMC8137566

[126] Adhikary S, Eilers M. Transcriptional regulation and transformation by Myc proteins. Nat Rev Mol Cell Biol. 2005;6(8):635–645. 16064138 10.1038/nrm1703

[127] Yu H, Shi T, Yao L, Xu D, Ding Y, Xia Q, Liu W, Wang X. Elevated nuclear PIGL disrupts the cMyc/BRD4 axis and improves PD-1 blockade therapy by dampening tumor immune evasion. Cell Mol Immunol. 2023;20(8):867–880. 37280393 10.1038/s41423-023-01048-3 PMC10387471

[128] Han H, Jain AD, Truica MI, Izquierdo-Ferrer J, Anker JF, Lysy B, Sagar V, Luan Y, Chalmers ZR, Unno K, et al. Small-molecule MYC inhibitors suppress tumor growth and enhance immunotherapy. Cancer Cell. 2019;36(5):483–497.e15. 31679823 10.1016/j.ccell.2019.10.001 PMC6939458

[129] Zhang W, Liu B, Wu W, Li L, Broom BM, Basourakos SP, Korentzelos D, Luan Y, Wang J, Yang G, et al. Targeting the MYCN-PARP-DNA damage response pathway in neuroendocrine prostate cancer. Clin Cancer Res. 2018;24(3):696–707. 29138344 10.1158/1078-0432.CCR-17-1872 PMC5823274

[130] Liang H, Yang C, Zeng R, Song Y, Wang J, Xiong W, Yan B, Jin X. Targeting CBX3 with a dual BET/PLK1 inhibitor enhances the antitumor efficacy of CDK4/6 inhibitors in prostate cancer. Adv Sci. 2023;24(3): Article e2302368. 10.1002/advs.202302368 PMC10754129 37949681

[131] Wu C, Peng S, Pilié PG, Geng C, Park S, Manyam GC, Lu Y, Yang G, Tang Z, Kondraganti S, et al. PARP and CDK4/6 inhibitor combination therapy induces apoptosis and suppresses neuroendocrine differentiation in prostate cancer. Mol Cancer Ther. 2021;20:1680–1691. 34158347 10.1158/1535-7163.MCT-20-0848 PMC8456452

[132] Sooreshjani MA, Kamra M, Zoubeidi A, Shah K. Reciprocal deregulation of NKX3.1 and AURKA axis in castration-resistant prostate cancer and NEPC models. J Biomed Sci. 2021;28(1):68. 34625072 10.1186/s12929-021-00765-z PMC8499580

[133] Gritsina G, Fong KW, Lu X, Lin Z, Xie W, Agarwal S, Lin D, Schiltz GE, Beltran H, Corey E, et al. Chemokine receptor CXCR7 activates Aurora kinase A and promotes neuroendocrine prostate cancer growth. J Clin Invest. 2023;133(15): Article e166248. 37347559 10.1172/JCI166248 PMC10378179

[134] Kufe D. Dependence on MUC1-C in progression of neuroendocrine prostate cancer. Int J Mol Sci. 2023;24(4):3719. 36835130 10.3390/ijms24043719 PMC9967814

[135] Kufe DW. Mucins in cancer: Function, prognosis and therapy. Nat Rev Cancer. 2009;9(12):874–885. 19935676 10.1038/nrc2761 PMC2951677

[136] Hagiwara M, Yasumizu Y, Yamashita N, Rajabi H, Fushimi A, Long MD, Li W, Bhattacharya A, Ahmad R, Oya M, et al. MUC1-C activates the BAF (mSWI/SNF) complex in prostate cancer stem cells. Cancer Res. 2021;81(4):1111–1122. 33323379 10.1158/0008-5472.CAN-20-2588 PMC8026569

[137] Kriplani P, Guarve K. Eudragit, a nifty polymer for anticancer preparations: A patent review. Recent Pat Anticancer Drug Discov. 2022;17(1):92–101. 34645379 10.2174/1574892816666211013113841

[138] Lee AR, Gan Y, Tang Y, Dong X. A novel mechanism of SRRM4 in promoting neuroendocrine prostate cancer development via a pluripotency gene network. EBioMedicine. 2018;35:167–177. 30100395 10.1016/j.ebiom.2018.08.011 PMC6154886

[139] Yamada Y, Venkadakrishnan VB, Mizuno K, Bakht M, Ku SY, Garcia MM, Beltran H. Targeting DNA methylation and B7-H3 in RB1-deficient and neuroendocrine prostate cancer. Sci Transl Med. 2023;15(722): Article eadf6732. 37967200 10.1126/scitranslmed.adf6732 PMC10954288

[140] Xie W, Agarwal S, Yu J. Ferroptosis: The vulnerability within a cancer monster. J Clin Invest. 2023;133(10): Article e170027. 37183818 10.1172/JCI170027 PMC10178832

[141] Mandigo AC, Yuan W, Xu K, Gallagher P, Pang A, Guan YF, Shafi AA, Thangavel C, Sheehan B, Bogdan D, et al. RB/E2F1 as a master regulator of cancer cell metabolism in advanced disease. Cancer Discov. 2021;11(9):2334–2353. 33879449 10.1158/2159-8290.CD-20-1114 PMC8419081

[142] Tan HL, Sood A, Rahimi HA, Wang W, Gupta N, Hicks J, Mosier S, Gocke CD, Epstein JI, Netto GJ, et al. Rb loss is characteristic of prostatic small cell neuroendocrine carcinoma. Clin Cancer Res. 2014;20(4):890–903. 24323898 10.1158/1078-0432.CCR-13-1982 PMC3931005

[143] Kumaraswamy A, Duan Z, Flores D, Zhang C, Sehrawat A, Hu YM, Swaim OA, Rodansky E, Storck WK, Kuleape JA, et al. LSD1 promotes prostate cancer cell reprogramming by repressing TP53 signaling independently of its demethylase function. JCI Insight. 2023;8(15): Article e167440. 37440313 10.1172/jci.insight.167440 PMC10445684

[144] Akamatsu S, Wyatt AW, Lin D, Lysakowski S, Zhang F, Kim S, Tse C, Wang K, Mo F, Haegert A, et al. The placental gene PEG10 promotes progression of neuroendocrine prostate cancer. Cell Rep. 2015;12(4):922–936. 26235627 10.1016/j.celrep.2015.07.012

[145] Liu B, Li L, Yang G, Geng C, Luo Y, Wu W, Manyam GC, Korentzelos D, Park S, Tang Z, et al. PARP inhibition suppresses GR-MYCN-CDK5-RB1-E2F1 signaling and neuroendocrine differentiation in castration-resistant prostate cancer. Clin Cancer Res. 2019;25(22):6839–6851. 31439587 10.1158/1078-0432.CCR-19-0317 PMC6858969

[146] Sekino Y, Pham QT, Kobatake K, Kitano H, Ikeda K, Goto K, Inoue S, Hayashi T, Shiota M, Yasui W, et al. HOXB5 overexpression is associated with neuroendocrine differentiation and poor prognosis in prostate cancer. Biomedicines. 2021;9(8):893. 34440097 10.3390/biomedicines9080893 PMC8389587

[147] Fan L, Gong Y, He Y, Gao WQ, Dong X, Dong B, Zhu HH, Xue W. TRIM59 is suppressed by androgen receptor and acts to promote lineage plasticity and treatment-induced neuroendocrine differentiation in prostate cancer. Oncogene. 2023;42(8):559–571. 36544044 10.1038/s41388-022-02498-1

[148] Yoshida M, Oda C, Mishima K, Tsuji I, Obika S, Shimojo M. An antisense amido-bridged nucleic acid gapmer oligonucleotide targeting SRRM4 alters REST splicing and exhibits anti-tumor effects in small cell lung cancer and prostate cancer cells. Cancer Cell Int. 2023;23(1):8. 36650528 10.1186/s12935-022-02842-1 PMC9847160

[149] Lee AR, Che N, Lovnicki JM, Dong X. Development of neuroendocrine prostate cancers by the Ser/Arg repetitive matrix 4-mediated RNA splicing network. Front Oncol. 2018;8:93. 29666783 10.3389/fonc.2018.00093 PMC5891588

[150] Lee AR, Gan Y, Xie N, Ramnarine VR, Lovnicki JM, Dong X. Alternative RNA splicing of the GIT1 gene is associated with neuroendocrine prostate cancer. Cancer Sci. 2019;110(1):245–255. 30417466 10.1111/cas.13869 PMC6317919

[151] Chen R, Li Y, Buttyan R, Dong X. Implications of PI3K/AKT inhibition on REST protein stability and neuroendocrine phenotype acquisition in prostate cancer cells. Oncotarget. 2017;8(47):84863–84876. 29156689 10.18632/oncotarget.19386 PMC5689579

[152] Mishima K, Obika S, Shimojo M. Splice-switching antisense oligonucleotide controlling tumor suppressor REST is a novel therapeutic medicine for neuroendocrine cancer. Mol Ther Nucleic Acids. 2024;35(3):102250. 39377066 10.1016/j.omtn.2024.102250 PMC11456559

[153] Shan J et al. Targeting Wnt/EZH2/microRNA-708 signaling pathway inhibits neuroendocrine differentiation in prostate cancer. Cell Death Discov. 2019;5:139. 31583122 10.1038/s41420-019-0218-y PMC6768854

[154] Luo J, Wang K, Yeh S, Sun Y, Liang L, Xiao Y, Xu W, Niu Y, Cheng L, Maity SN, et al. LncRNA-p21 alters the antiandrogen enzalutamide-induced prostate cancer neuroendocrine differentiation via modulating the EZH2/STAT3 signaling. Nat Commun. 2019;10(1):2571. 31189930 10.1038/s41467-019-09784-9 PMC6561926

[155] Chen L, Ji Y, Li A, Liu B, Shen K, Su R, Ma Z, Zhang W, Wang Q, Zhu Y, et al. High-throughput drug screening identifies fluoxetine as a potential therapeutic agent for neuroendocrine prostate cancer. Front Oncol. 2023;13:1085569. 36994207 10.3389/fonc.2023.1085569 PMC10042075

[156] Yin Y, Xu L, Chang Y, Zeng T, Chen X, Wang A, Groth J, Foo WC, Liang C, Hu H, et al. N-Myc promotes therapeutic resistance development of neuroendocrine prostate cancer by differentially regulating miR-421/ATM pathway. Mol Cancer. 2019;18(1):11. 30657058 10.1186/s12943-019-0941-2 PMC6337850

[157] Fernandes RC, Toubia J, Townley S, Hanson AR, Dredge BK, Pillman KA, Bert AG, Winter JM, Iggo R, das R, et al. Post-transcriptional gene regulation by microRNA-194 promotes neuroendocrine transdifferentiation in prostate cancer. Cell Rep. 2021;34(1):108585. 33406413 10.1016/j.celrep.2020.108585

[158] Baca SC, Takeda DY, Seo JH, Hwang J, Ku SY, Arafeh R, Arnoff T, Agarwal S, Bell C, O’Connor E, et al. Reprogramming of the FOXA1 cistrome in treatment-emergent neuroendocrine prostate cancer. Nat Commun. 2021;12(1):1979. 33785741 10.1038/s41467-021-22139-7 PMC8010057

[159] Kim J, Jin H, Zhao JC, Yang YA, Li Y, Yang X, Dong X, Yu J. FOXA1 inhibits prostate cancer neuroendocrine differentiation. Oncogene. 2017;36(28):4072–4080. 28319070 10.1038/onc.2017.50 PMC5509480

[160] Masone MC. FOXA2-KIT-driven lineage plasticity in NEPC. Nat Rev Urol. 2023;20(1):8. 10.1038/s41585-022-00694-y 36477218

[161] Lee AR, Li Y, Xie N, Gleave ME, Cox ME, Collins CC, Dong X. Alternative RNA splicing of the MEAF6 gene facilitates neuroendocrine prostate cancer progression. Oncotarget. 2017;8(17):27966–27975. 28427194 10.18632/oncotarget.15854 PMC5438622

[162] Li H, Wang L, Li Z, Geng X, Li M, Tang Q, Wu C, Lu Z. SOX2 has dual functions as a regulator in the progression of neuroendocrine prostate cancer. Lab Investig. 2020;100(40):570–582. 31772313 10.1038/s41374-019-0343-5

[163] Fraser JA, Sutton JE, Tazayoni S, Bruce I, Poole AV. hASH1 nuclear localization persists in neuroendocrine transdifferentiated prostate cancer cells, even upon reintroduction of androgen. Sci Rep. 2019;9(1):19076. 31836808 10.1038/s41598-019-55665-y PMC6911083

[164] Koh J, Kim H, Moon KC, Lee C, Lee K, Ryu HS, Jung KC, Jeon YK. Molecular classification of extrapulmonary neuroendocrine carcinomas with emphasis on POU2F3-positive tuft cell carcinoma. Am J Surg Pathol. 2023;47(2):183–193. 36253891 10.1097/PAS.0000000000001977 PMC9833113

[165] Guo H, Ci X, Ahmed M, Hua JT, Soares F, Lin D, Puca L, Vosoughi A, Xue H, Li E, et al. ONECUT2 is a driver of neuroendocrine prostate cancer. Nat Commun. 2019;10(1):278. 30655535 10.1038/s41467-018-08133-6 PMC6336817

[166] Li W, Sun X. Recent advances in developing novel anti-cancer drugs targeting tumor hypoxic and acidic microenvironments. Recent Pat Anticancer Drug Discov. 2018;13(4):455–468. 30173649 10.2174/1574892813666180831102519

[167] Rotinen M, You S, Yang J, Coetzee SG, Reis-Sobreiro M, Huang WC, Huang F, Pan X, Yáñez A, Hazelett DJ, et al. ONECUT2 is a targetable master regulator of lethal prostate cancer that suppresses the androgen axis. Nat Med. 2018;24(12):1887–1898. 30478421 10.1038/s41591-018-0241-1 PMC6614557

[168] Meng F, Evans JW, Bhupathi D, Banica M, Lan L, Lorente G, Duan JX, Cai X, Mowday AM, Guise CP, et al. Molecular and cellular pharmacology of the hypoxia-activated prodrug TH-302. Mol Cancer Ther. 2012;11(3):740–751. 22147748 10.1158/1535-7163.MCT-11-0634

[169] VanDeusen HR, Ramroop JR, Morel KL, Bae SY, Sheahan AV, Sychev Z, Lau NA, Cheng LC, Tan VM, Li Z, et al. Targeting RET kinase in neuroendocrine prostate cancer. Mol Cancer Res. 2020;18(8):1176–1188. 32461304 10.1158/1541-7786.MCR-19-1245 PMC7415621

[170] Cyrta J, Augspach A, de Filippo MR, Prandi D, Thienger P, Benelli M, Cooley V, Bareja R, Wilkes D, Chae SS, et al. Role of specialized composition of SWI/SNF complexes in prostate cancer lineage plasticity. Nat Commun. 2020;11(1):5549. 33144576 10.1038/s41467-020-19328-1 PMC7642293

[171] Li JJ, Vasciaveo A, Karagiannis D, Sun Z, Chen X, Socciarelli F, Frankenstein Z, Zou M, Pannellini T, Chen Y, et al. NSD2 maintains lineage plasticity and castration-resistance in neuroendocrine prostate cancer. bioRxiv. 2023. 10.1101/2023.07.18.549585.

[172] Mosquera MJ, Kim S, Bareja R, Fang Z, Cai S, Pan H, Asad M, Martin ML, Sigouros M, Rowdo FM, et al. Extracellular matrix in synthetic hydrogel-based prostate cancer organoids regulate therapeutic response to EZH2 and DRD2 inhibitors. Adv Mater. 2022;34(2): Article e2100096. 34676924 10.1002/adma.202100096 PMC8820841

[173] Wang J, Xu LF, Liu C, Huang T, Liang CZ, Fan YD. Identifying the role of apolipoprotein A-I in prostate cancer. Asian J Androl. 2021;23(4):400–408. 33586698 10.4103/aja.aja_92_20 PMC8269822

[174] Xing P, Wang S, Cao Y, Liu B, Zheng F, Guo W, Huang J, Zhao Z, Yang Z, Lin X, et al. Treatment strategies and drug resistance mechanisms in adenocarcinoma of different organs. Drug Resist Updat. 2023;71:101002. 37678078 10.1016/j.drup.2023.101002

[175] Wang H, Liu J, Zhu X, Yang B, He Z, Yao X. AZGP1P2/UBA1/RBM15 cascade mediates the fate determinations of prostate cancer stem cells and promotes therapeutic effect of docetaxel in castration-resistant prostate cancer via TPM1 m6A modification. Research. 2023;6:0252. 37854295 10.34133/research.0252 PMC10581371

[176] Chen L, Huang M. Oncometabolites in cancer: From cancer cells to the tumor microenvironment. Holist Integr Oncol. 2024;3:26.

[177] Mo F, Lin D, Takhar M, Ramnarine VR, Dong X, Bell RH, Volik SV, Wang K, Xue H, Wang Y, et al. Stromal gene expression is predictive for metastatic primary prostate cancer. Eur Urol. 2018;73(4):524–532. 28330676 10.1016/j.eururo.2017.02.038 PMC6685211

[178] Kim IS, Zhang XH. One microenvironment does not fit all: Heterogeneity beyond cancer cells. Cancer Metastasis Rev. 2016;35(4):601–629. 27858305 10.1007/s10555-016-9643-z PMC5215976

[179] Long X, Hou H, Wang X, Liu S, Diao T, Lai S, Hu M, Zhang S, Liu M, Zhang H. Immune signature driven by ADT-induced immune microenvironment remodeling in prostate cancer is correlated with recurrence-free survival and immune infiltration. Cell Death Dis. 2020;11(9):779. 32951005 10.1038/s41419-020-02973-1 PMC7502080

[180] Paranjape AN, Soundararajan R, Werden SJ, Joseph R, Taube JH, Liu H, Rodriguez-Canales J, Sphyris N, Wistuba I, Miura N, et al. Inhibition of FOXC2 restores epithelial phenotype and drug sensitivity in prostate cancer cells with stem-cell properties. Oncogene. 2016;35(46):5963–5976. 26804168 10.1038/onc.2015.498 PMC5116559

[181] Lee GT, Kwon SJ, Lee JH, Jeon SS, Jang KT, Choi HY, Lee HM, Kim WJ, Lee DH, Kim IY. Macrophages induce neuroendocrine differentiation of prostate cancer cells via BMP6-IL6 loop. Prostate. 2011;71(14):1525–1537. 21374653 10.1002/pros.21369

[182] Jeannin P, Paolini L, Adam C, Delneste Y. The roles of CSFs on the functional polarization of tumor-associated macrophages. FEBS J. 2018;285(4):680–699. 29171156 10.1111/febs.14343

[183] Wang C, Peng G, Huang H, Liu F, Kong DP, Dong KQ, Dai LH, Zhou Z, Wang KJ, Yang J, et al. Blocking the feedback loop between neuroendocrine differentiation and macrophages improves the therapeutic effects of enzalutamide (MDV3100) on prostate cancer. Clin Cancer Res. 2018;24(3):708–723. 29191973 10.1158/1078-0432.CCR-17-2446

[184] Kohada Y, Kaiho Y, Takeda K, Kuromoto A, Ito J, Teishima J, Nakamura Y, Kaifu T, Nakamura A, Sato M. Analysis of the circulating myeloid-derived suppressor cells during androgen deprivation therapy for prostate cancer. IJU Case Rep. 2021;4(6):367–370. 34755058 10.1002/iju5.12351 PMC8560438

[185] Myeloid-derived suppressor cells in prostate cancer: Present knowledge and future perspectives. Cells. 2021;11(1):20. 35011582 10.3390/cells11010020 PMC8750906

[186] Roca H, Varsos ZS, Sud S, Craig MJ, Ying C, Pienta KJ. CCL2 and interleukin-6 promote survival of human CD11b+ peripheral blood mononuclear cells and induce M2-type macrophage polarization. J Biol Chem. 2009;284(49):34342–34354. 19833726 10.1074/jbc.M109.042671 PMC2797202

[187] Heusinkveld M, de Vos van Steenwijk PJ, Goedemans R, Ramwadhdoebe TH, Gorter A, Welters MJP, van Hall T, van der Burg SH. M2 macrophages induced by prostaglandin E2 and IL-6 from cervical carcinoma are switched to activated M1 macrophages by CD4+ Th1 cells. J Immunol. 2011;187(3):1157–1165. 21709158 10.4049/jimmunol.1100889

[188] Bonollo F, Thalmann GN, Kruithof-de Julio M, Karkampouna S. The role of cancer-associated fibroblasts in prostate cancer tumorigenesis. Cancers. 2020;12(7):1887. 32668821 10.3390/cancers12071887 PMC7409163

[189] Nguyen EV, Pereira BA, Lawrence MG, Ma X, Rebello RJ, Chan H, Niranjan B, Wu Y, Ellem S, Guan X, et al. Proteomic profiling of human prostate cancer-associated fibroblasts (CAF) reveals LOXL_2_-dependent regulation of the tumor microenvironment. Mol Cell Proteomics. 2019;18(7):1410–1427. 31061140 10.1074/mcp.RA119.001496 PMC6601211

[190] Yu Y, Zhang Q, Ma C, Yang X, Lin R, Zhang H, Liu Y, Han Z, Cheng J. Mesenchymal stem cells recruited by castration-induced inflammation activation accelerate prostate cancer hormone resistance via chemokine ligand 5 secretion. Stem Cell Res Ther. 2018;9(1):242. 30257726 10.1186/s13287-018-0989-8 PMC6158918

[191] Yu Y, Yang FH, Zhang WT, Guo YD, Ye L, Yao XD. Mesenchymal stem cells desensitize castration-resistant prostate cancer to docetaxel chemotherapy via inducing TGF-β1-mediated cell autophagy. Cell Biosci. 2021;11(1):7. 33413648 10.1186/s13578-020-00494-0 PMC7792182

[192] von Hardenberg J, Hartmann S, Nitschke K, Worst TS, Ting S, Reis H, Nuhn P, Weis CA, Erben P. Programmed death ligand 1 (PD-L1) status and tumor-infiltrating lymphocytes in hot spots of primary and liver metastases in prostate cancer with neuroendocrine differentiation. *Clin Genitourin Cancer*. 2019;**17**(2):145–153.e5. 10.1016/j.clgc.2018.12.007 30709785

[193] Baek D-S, Kim Y-J, Vergara S, Conard A, Adams C, Calero G, Ishima R, Mellors JW, Dimitrov DS. A highly-specific fully-human antibody and CAR-T cells targeting CD66e/CEACAM5 are cytotoxic for CD66e-expressing cancer cells in vitro and in vivo. Cancer Lett. 2022;525:97–107. 34740610 10.1016/j.canlet.2021.10.041

[194] Zetrini AE, Lip HY, Abbasi AZ, Alradwan I, Ahmed T, He C, Henderson JT, Rauth AM, Wu XY. Remodeling tumor immune microenvironment by using polymer-lipid-manganese dioxide nanoparticles with radiation therapy to boost immune response of castration-resistant prostate cancer. Research. 2023;6:0247. 37795337 10.34133/research.0247 PMC10546607

[195] Lee EC, Frolov A, Li R, Ayala G, Greenberg NM. Targeting Aurora kinases for the treatment of prostate cancer. Cancer Res. 2006;66(10):4996–5002. 16707419 10.1158/0008-5472.CAN-05-2796

[196] Sun F, Zhang ZW, Tan EM, Lim ZLR, Li Y, Wang XC, Chua SE, Li J, Cheung E, Yong EL. Icaritin suppresses development of neuroendocrine differentiation of prostate cancer through inhibition of IL-6/STAT3 and Aurora kinase A pathways in TRAMP mice. Carcinogenesis. 2016;37(7):701–711. 27207661 10.1093/carcin/bgw044

[197] Li Y, Xie N, Chen R, Lee AR, Lovnicki J, Morrison EA, Fazli L, Zhang Q, Musselman CA, Wang Y, et al. RNA splicing of the BHC80 gene contributes to neuroendocrine prostate cancer progression. Eur Urol. 2019;76(2):157–166. 30910347 10.1016/j.eururo.2019.03.011

[198] Hotte SJ, Chi KN, Joshua AM, Tu D, Macfarlane RJ, Gregg RW, Ruether JD, Basappa NS, Finch D, Salim M, et al. A phase II study of PX-866 in patients with recurrent or metastatic castration-resistant prostate cancer: Canadian cancer trials group study IND205. Clin Genitourin Cancer. 2019;17(3):201–208.e201. 31056399 10.1016/j.clgc.2019.03.005

[199] Cortés MA, Cariaga-Martinez AE, Lobo MV, Martín Orozco RM, Motiño O, Rodríguez-Ubreva FJ, Angulo J, López-Ruiz P, Colás B. EGF promotes neuroendocrine-like differentiation of prostate cancer cells in the presence of LY294002 through increased ErbB2 expression independent of the phosphatidylinositol 3-kinase-AKT pathway. Carcinogenesis. 2012;33(6):1169–1177. 22461520 10.1093/carcin/bgs139

[200] Sweeney C, Bracarda S, Sternberg CN, Chi KN, Olmos D, Sandhu S, Massard C, Matsubara N, Alekseev B, Parnis F, et al. Ipatasertib plus abiraterone and prednisolone in metastatic castration-resistant prostate cancer (IPATential150): A multicentre, randomised, double-blind, phase 3 trial. Lancet. 2021;398(10295):131–142. 34246347 10.1016/S0140-6736(21)00580-8

[201] Aggarwal RR, Schweizer MT, Nanus DM, Pantuck AJ, Heath EI, Campeau E, Attwell S, Norek K, Snyder M, Bauman L, et al. A phase Ib/IIa study of the pan-BET inhibitor ZEN-3694 in combination with enzalutamide in patients with metastatic castration-resistant prostate cancer. Clin Cancer Res. 2020;26(20):5338–5347. 32694156 10.1158/1078-0432.CCR-20-1707 PMC7572827

[202] Kim DH, Sun D, Storck WK, Welker Leng K, Jenkins C, Coleman DJ, Sampson D, Guan X, Kumaraswamy A, Rodansky ES, et al. BET bromodomain inhibition blocks an AR-repressed, E2F1-activated treatment-emergent neuroendocrine prostate cancer lineage plasticity program. Clin Cancer Res. 2021;27(17):4923–4936. 34145028 10.1158/1078-0432.CCR-20-4968 PMC8416959

[203] Boike L, Cioffi AG, Majewski FC, Co J, Henning NJ, Jones MD, Liu G, McKenna JM, Tallarico JA, Schirle M, et al. Discovery of a functional covalent ligand targeting an intrinsically disordered cysteine within MYC. Cell Chem Biol. 2021;28(1):4–13.e17. 32966806 10.1016/j.chembiol.2020.09.001 PMC7854864

[204] Ton AT, Foo J, Singh K, Lee J, Kalyta A, Morin H, Perez C, Ban F, Leblanc E, Lallous N, et al. Development of VPC-70619, a small-molecule N-Myc inhibitor as a potential therapy for neuroendocrine prostate cancer. Int J Mol Sci. 2022;23(5):2588. 35269731 10.3390/ijms23052588 PMC8910697

[205] Hart JR, Garner AL, Yu J, Ito Y, Sun M, Ueno L, Rhee JK, Baksh MM, Stefan E, Hartl M, et al. Inhibitor of MYC identified in a Krohnke pyridine library. Proc Natl Acad Sci USA. 2014;111(34):12556–12561. 25114221 10.1073/pnas.1319488111 PMC4151726

[206] Singh A, Kumar A, Kumar P, Nayak N, Bhardwaj T, Giri R, Garg N. A novel inhibitor L755507 efficiently blocks c-Myc-MAX heterodimerization and induces apoptosis in cancer cells. J Biol Chem. 2021;297:100903. 34157284 10.1016/j.jbc.2021.100903 PMC8294579

[207] Blanc C, Moktefi A, Jolly A, de la Grange P, Gay D, Nicolaiew N, Semprez F, Maillé P, Soyeux P, Firlej V, et al. The Neuropilin-1/PKC axis promotes neuroendocrine differentiation and drug resistance of prostate cancer. Br J Cancer. 2023;128(5):918–927. 36550208 10.1038/s41416-022-02114-9 PMC9977768

[208] Hasegawa M, Sinha RK, Kumar M, Alam M, Yin L, Raina D, Kharbanda A, Panchamoorthy G, Gupta D, Singh H, et al. Intracellular targeting of the oncogenic MUC1-C protein with a novel GO-203 nanoparticle formulation. Clin Cancer Res. 2015;21(10):2338–2347. 25712682 10.1158/1078-0432.CCR-14-3000 PMC4433879

[209] Panchamoorthy G, Jin C, Raina D, Bharti A, Yamamoto M, Adeebge D, Zhao Q, Bronson R, Jiang S, Li L, et al. Targeting the human MUC1-C oncoprotein with an antibody-drug conjugate. JCI Insight. 2018;3(12): Article e99880. 29925694 10.1172/jci.insight.99880 PMC6124453

[210] Sehrawat A, Gao L, Wang Y, Bankhead A 3rd, McWeeney S, King CJ, Schwartzman J, Urrutia J, Bisson WH, Coleman DJ, et al. LSD1 activates a lethal prostate cancer gene network independently of its demethylase function. Proc Natl Acad Sci USA. 2018;115(18):E4179–E4188. 29581250 10.1073/pnas.1719168115 PMC5939079

[211] Hollebecque A, Salvagni S, Plummer R, Niccoli P, Capdevila J, Curigliano G, Moreno V, de Braud F, de Villambrosia SG, Martin-Romano P, et al. Clinical activity of CC-90011, an oral, potent, and reversible LSD1 inhibitor, in advanced malignancies. Cancer. 2022;128(17):3185–3195. 35737639 10.1002/cncr.34366 PMC9540525

[212] Natani S, Sruthi KK, Asha SM, Khilar P, Lakshmi PSV, Ummanni R. Activation of TGF-beta - SMAD2 signaling by IL-6 drives neuroendocrine differentiation of prostate cancer through p38MAPK. Cell Signal. 2022;91:110240. 34986386 10.1016/j.cellsig.2021.110240

[213] Zhao X, Zhou T, Wang Y, Bao M, Ni C, Ding L, Sun S, Dong H, Li J, Liang C. Trigred motif 36 regulates neuroendocrine differentiation of prostate cancer via HK2 ubiquitination and GPx4 deficiency. Cancer Sci. 2023;114(6):2445–2459. 36799474 10.1111/cas.15763 PMC10236700

[214] Wang W, Wang M, du T, Hou Z, You S, Zhang S, Ji M, Xue N, Chen X. SHMT2 promotes gastric cancer development through regulation of HIF1α/VEGF/STAT3 signaling. Int J Mol Sci. 2023;24(8):7150. 37108312 10.3390/ijms24087150 PMC10138966

[215] Kroon P, Berry PA, Stower MJ, Rodrigues G, Mann VM, Simms M, Bhasin D, Chettiar S, Li C, Li PK, et al. JAK-STAT blockade inhibits tumor initiation and clonogenic recovery of prostate cancer stem-like cells. Cancer Res. 2013;73(16):5288–5298. 23824741 10.1158/0008-5472.CAN-13-0874

[216] Dorff TB, Goldman B, Pinski JK, Mack PC, Lara PN Jr, van Veldhuizen PJ Jr, Quinn DI, Vogelzang NJ, Thompson IM Jr, Hussain MHA. Clinical and correlative results of SWOG S0354: A phase II trial of CNTO328 (siltuximab), a monoclonal antibody against interleukin-6, in chemotherapy-pretreated patients with castration-resistant prostate cancer. Clin Cancer Res. 2010;16(11):3028–3034. 20484019 10.1158/1078-0432.CCR-09-3122 PMC2898710

[217] Hellsten R, Johansson M, Dahlman A, Dizeyi N, Sterner O, Bjartell A. Galiellalactone is a novel therapeutic candidate against hormone-refractory prostate cancer expressing activated Stat3. Prostate. 2008;68(3):269–280. 18163422 10.1002/pros.20699

[218] Gan Y, Li Y, Long Z, Lee AR, Xie N, Lovnicki JM, Tang Y, Chen X, Huang J, Dong X. Roles of alternative RNA splicing of the Bif-1 gene by SRRM4 during the development of treatment-induced neuroendocrine prostate cancer. EBioMedicine. 2018;31:267–275. 29759485 10.1016/j.ebiom.2018.05.002 PMC6013970

[219] Coleman DJ, Sampson DA, Sehrawat A, Kumaraswamy A, Sun D, Wang Y, Schwartzman J, Urrutia J, Lee AR, Coleman IM, et al. Alternative splicing of LSD1+8a in neuroendocrine prostate cancer is mediated by SRRM4. Neoplasia. 2020;22(8):253–262. 32403054 10.1016/j.neo.2020.04.002 PMC7218227

[220] Naderinezhad S, Zhang G, Wang Z, Zheng D, Hulsurkar M, Bakhoum M, Su N, Yang H, Shen T, Li W. A novel GRK3-HDAC2 regulatory pathway is a key direct link between neuroendocrine differentiation and angiogenesis in prostate cancer progression. Cancer Lett. 2023;571:216333. 37543278 10.1016/j.canlet.2023.216333 PMC11235056

[221] Tiwari R, Manzar N, Bhatia V, Yadav A, Nengroo MA, Datta D, Carskadon S, Gupta N, Sigouros M, Khani F, et al. Androgen deprivation upregulates SPINK1 expression and potentiates cellular plasticity in prostate cancer. Nat Commun. 2020;11(1):384. 31959826 10.1038/s41467-019-14184-0 PMC6971084

[222] Lewis CS, Voelkel-Johnson C, Smith CD. Targeting sphingosine kinases for the treatment of cancer. Adv Cancer Res. 2018;140:295–325. 30060814 10.1016/bs.acr.2018.04.015 PMC6447312

[223] Chen SY, Chen KL, Ding LY, Yu CH, Wu HY, Chou YY, Chang CJ, Chang CH, Wu YN, Wu SR, et al. RNA bisulfite sequencing reveals NSUN2-mediated suppression of epithelial differentiation in pancreatic cancer. Oncogene. 2022;41(22):3162–3176. 35501460 10.1038/s41388-022-02325-7

[224] Ci X, Hao J, Dong X, Choi SY, Xue H, Wu R, Qu S, Gout PW, Zhang F, Haegert AM, et al. Heterochromatin protein 1alpha mediates development and aggressiveness of neuroendocrine prostate cancer. Cancer Res. 2018;78(10):2691–2704. 29487201 10.1158/0008-5472.CAN-17-3677

[225] Zhang Y, Zheng D, Zhou T, Song H, Hulsurkar M, Su N, Liu Y, Wang Z, Shao L, Ittmann M, et al. Androgen deprivation promotes neuroendocrine differentiation and angiogenesis through CREB-EZH2-TSP1 pathway in prostate cancers. Nat Commun. 2018;9(1):4080. 30287808 10.1038/s41467-018-06177-2 PMC6172226

[226] Bland T, Wang J, Yin L, Pu T, Li J, Gao J, Lin TP, Gao AC, Wu BJ. WLS-Wnt signaling promotes neuroendocrine prostate cancer. iScience. 2021;24(1):101970. 33437943 10.1016/j.isci.2020.101970 PMC7788232

[227] Song Z, Cao Q, Guo B, Zhao Y, Li X, Lou N, Zhu C, Luo G, Peng S, Li G, et al. Overexpression of RACGAP1 by E2F1 promotes neuroendocrine differentiation of prostate cancer by stabilizing EZH2 expression. Aging Dis. 2023;24(1):101970. 10.14336/AD.2023.0202 PMC10529746 37196108

[228] Long Z, Deng L, Li C, He Q, He Y, Hu X, Cai Y, Gan Y. Loss of EHF facilitates the development of treatment-induced neuroendocrine prostate cancer. Cell Death Dis. 2021;12(1):46. 33414441 10.1038/s41419-020-03326-8 PMC7790822

[229] Beltran H, Prandi D, Mosquera JM, Benelli M, Puca L, Cyrta J, Marotz C, Giannopoulou E, Chakravarthi BVSK, Varambally S, et al. Divergent clonal evolution of castration-resistant neuroendocrine prostate cancer. Nat Med. 2016;22(3):298–305. 26855148 10.1038/nm.4045 PMC4777652

[230] Dang Q, Li L, Xie H, He D, Chen J, Song W, Chang LS, Chang HC, Yeh S, Chang C. Anti-androgen enzalutamide enhances prostate cancer neuroendocrine (NE) differentiation via altering the infiltrated mast cells --> androgen receptor (AR) --> miRNA32 signals. Mol Oncol. 2015;9(7):1241–1251. 25817444 10.1016/j.molonc.2015.02.010 PMC5528811

[231] Natani S, Ramakrishna M, Nallavolu T, Ummanni R. MicroRNA-147b induces neuroendocrine differentiation of prostate cancer cells by targeting ribosomal protein RPS15A. Prostate. 2023;83(10):936–949. 37069746 10.1002/pros.24535

[232] Mather RL, Parolia A, Carson SE, Venalainen E, Roig-Carles D, Jaber M, Chu SC, Alborelli I, Wu R, Lin D, et al. The evolutionarily conserved long non-coding RNA LINC00261 drives neuroendocrine prostate cancer proliferation and metastasis via distinct nuclear and cytoplasmic mechanisms. Mol Oncol. 2021;15(7):1921–1941. 33793068 10.1002/1878-0261.12954 PMC8253100

[233] Liu Q, Pang J, Wang LA, Huang Z, Xu J, Yang X, Xie Q, Huang Y, Tang T, Tong D, et al. Histone demethylase PHF8 drives neuroendocrine prostate cancer progression by epigenetically upregulating FOXA2. J Pathol. 2021;253(1):106–118. 33009820 10.1002/path.5557 PMC7756255

[234] Yadav SS, Li J, Stockert JA, Herzog B, O'Connor J, Garzon-Manco L, Parsons R, Tewari AK, Yadav KK. Induction of neuroendocrine differentiation in prostate cancer cells by dovitinib (TKI-258) and its therapeutic implications. Transl Oncol. 2017;10(3):357–366. 28342996 10.1016/j.tranon.2017.01.011 PMC5369368

[235] Sonpavde GP, Pond GR, Fizazi K, de Bono JS, Basch EM, Scher HI, Smith MR. Cabozantinib for progressive metastatic castration-resistant prostate cancer following docetaxel: Combined analysis of two phase 3 trials. Eur Urol Oncol. 2020;3:540–543. 31412002 10.1016/j.euo.2018.11.006 PMC8428772

[236] Corn PG, Zhang M, Nogueras-Gonzalez GM, Xiao L, Zurita AJ, Subudhi SK, Tu SM, Aparicio AM, Coarfa C, Rajapakshe K, et al. A phase II study of cabozantinib and androgen ablation in patients with hormone-naive metastatic prostate cancer. Clin Cancer Res. 2020;26(5):990–999. 31941830 10.1158/1078-0432.CCR-19-2389

[237] Smith MR, Sweeney CJ, Corn PG, Rathkopf DE, Smith DC, Hussain M, George DJ, Higano CS, Harzstark AL, Sartor AO, et al. Cabozantinib in chemotherapy-pretreated metastatic castration-resistant prostate cancer: Results of a phase II nonrandomized expansion study. J Clin Oncol. 2014;32(30):3391–3399. 25225437 10.1200/JCO.2013.54.5954 PMC4383838

[238] Yan T, Zhou D, Shi Y, Cui D, Jiang J, Han B, Xia S, Wang Z, Liu H, Guo W, et al. Targeting ADT-induced activation of the E3 ubiquitin ligase Siah2 to delay the occurrence of castration-resistant prostate cancer. Front Oncol. 2021;11: Article 637040. 33937036 10.3389/fonc.2021.637040 PMC8085430

[239] Feng Y, Sessions EH, Zhang F, Ban F, Placencio-Hickok V, Ma CT, Zeng FY, Pass I, Terry DB, Cadwell G, et al. Identification and characterization of small molecule inhibitors of the ubiquitin ligases Siah1/2 in melanoma and prostate cancer cells. Cancer Lett. 2019;449:145–162. 30771432 10.1016/j.canlet.2019.02.012 PMC6411447

[240] Park SI, Zhang J, Phillips KA, Araujo JC, Najjar AM, Volgin AY, Gelovani JG, Kim SJ, Wang Z, Gallick GE. Targeting SRC family kinases inhibits growth and lymph node metastases of prostate cancer in an orthotopic nude mouse model. Cancer Res. 2008;68(9):3323–3333. 18451159 10.1158/0008-5472.CAN-07-2997

[241] Yu EY, Wilding G, Posadas E, Gross M, Culine S, Massard C, Morris MJ, Hudes G, Calabrò F, Cheng S, et al. Phase II study of dasatinib in patients with metastatic castration-resistant prostate cancer. Clin Cancer Res. 2009;15(23):7421–7428. 19920114 10.1158/1078-0432.CCR-09-1691 PMC3394097

[242] Twardowski PW, Beumer JH, Chen CS, Kraft AS, Chatta GS, Mitsuhashi M, Ye W, Christner SM, Lilly MB. A phase II trial of dasatinib in patients with metastatic castration-resistant prostate cancer treated previously with chemotherapy. Anti-Cancer Drugs. 2013;24(7):743–753. 23652277 10.1097/CAD.0b013e328361feb0 PMC4165488

[243] Araujo JC, Trudel GC, Saad F, Armstrong AJ, Yu EY, Bellmunt J, Wilding G, McCaffrey J, Serrano SV, Matveev VB, et al. Docetaxel and dasatinib or placebo in men with metastatic castration-resistant prostate cancer (READY): A randomised, double-blind phase 3 trial. Lancet Oncol. 2013;14(13):1307–1316. 24211163 10.1016/S1470-2045(13)70479-0 PMC5478530

[244] Jin XF, Spottl G, Maurer J, Nolting S, Auernhammer CJ. Antitumoral activity of the MEK inhibitor trametinib (TMT212) alone and in combination with the CDK4/6 inhibitor ribociclib (LEE011) in neuroendocrine tumor cells in vitro. Cancers. 2021;13(6):1495. 33807122 10.3390/cancers13061485 PMC8004919

[245] Lovnicki J, Gan Y, Feng T, Li Y, Xie N, Ho CH, Lee AR, Chen X, Nappi L, Han B, et al. LIN28B promotes the development of neuroendocrine prostate cancer. J Clin Invest. 2020;130:5338–5348. 32634132 10.1172/JCI135373 PMC7524485

[246] Radaeva M, Ho CH, Xie N, Zhang S, Lee J, Liu L, Lallous N, Cherkasov A, Dong X. Discovery of novel Lin28 inhibitors to suppress cancer cell stemness. Cancers. 2022;14(22):5687. 36428779 10.3390/cancers14225687 PMC9688808

[247] Wang J, Li J, Yin L, Pu T, Wei J, Karthikeyan V, Lin TP, Gao AC, Wu BJ. Discovery of novel Lin28 inhibitors to suppress cancer cell stemness. Oncogene. 2022;41(22):4307–4317.35986103

[248] Patel GK, Dutta S, Mahmud Syed M, Ramachandran S, Sharma M, Rajamanickam V, Ganapathy V, DeGraff D, Pruitt K, Tripathi M, et al. TBX2 drives neuroendocrine prostate cancer through exosome-mediated repression of miR-200c-3p. Cancers. 2021;13(19):5020. 34638504 10.3390/cancers13195020 PMC8507954

[249] Singh N, Ramnarine VR, Song JH, Pandey R, Padi SKR, Nouri M, Olive V, Kobelev M, Okumura K, McCarthy D, et al. The long noncoding RNA H19 regulates tumor plasticity in neuroendocrine prostate cancer. Nat Commun. 2021;12(1):7349. 34934057 10.1038/s41467-021-26901-9 PMC8692330

[250] Liu YN, Niu S, Chen WY, Zhang Q, Tao Y, Chen WH, Jiang KC, Chen X, Shi H, Liu A, et al. Leukemia inhibitory factor promotes castration-resistant prostate cancer and neuroendocrine differentiation by activated ZBTB46. Clin Cancer Res. 2019;25(13):4128–4140. 30962287 10.1158/1078-0432.CCR-18-3239 PMC7168873

[251] Chen WY, Zeng T, Wen YC, Yeh HL, Jiang KC, Chen WH, Zhang Q, Huang J, Liu YN. Androgen deprivation-induced ZBTB46-PTGS1 signaling promotes neuroendocrine differentiation of prostate cancer. Cancer Lett. 2019;440-441:35–46. 30312731 10.1016/j.canlet.2018.10.004

[252] Wang S, Alpsoy A, Sood S, Ordonez-Rubiano SC, Dhiman A, Sun Y, Jiao G, Krusemark CJ, Dykhuizen EC. A potent, selective CBX2 chromodomain ligand and its cellular activity during prostate cancer neuroendocrine differentiation. Chembiochem. 2021;22(13):2335–2344. 33950564 10.1002/cbic.202100118 PMC8358665

[253] Aytes A, Giacobbe A, Mitrofanova A, Ruggero K, Cyrta J, Arriaga J, Palomero L, Farran-Matas S, Rubin MA, Shen MM, et al. NSD2 is a conserved driver of metastatic prostate cancer progression. Nat Commun. 2018;9(1):5201. 30518758 10.1038/s41467-018-07511-4 PMC6281610

[254] Baritaki S, Yeung K, Palladino M, Berenson J, Bonavida B. Pivotal roles of snail inhibition and RKIP induction by the proteasome inhibitor NPI-0052 in tumor cell chemoimmunosensitization. Cancer Res. 2009;69(21):8376–8385. 19843864 10.1158/0008-5472.CAN-09-1069

[255] Baritaki S, Chapman A, Yeung K, Spandidos DA, Palladino M, Bonavida B. Inhibition of epithelial to mesenchymal transition in metastatic prostate cancer cells by the novel proteasome inhibitor, NPI-0052: Pivotal roles of snail repression and RKIP induction. Oncogene. 2009;28(40):3573–3585. 19633685 10.1038/onc.2009.214

[256] Mickova A, Kharaishvili G, Kurfurstova D, Gachechiladze M, Kral M, Vacek O, Pokryvkova B, Mistrik M, Soucek K, Bouchal J. Skp2 and Slug are coexpressed in aggressive prostate cancer and inhibited by neddylation blockade. Int J Mol Sci. 2021;22(6):2844. 33799604 10.3390/ijms22062844 PMC8000894

[257] Weissenrieder JS, Reilly JE, Neighbors JD, Hohl RJ. Inhibiting geranylgeranyl diphosphate synthesis reduces nuclear androgen receptor signaling and neuroendocrine differentiation in prostate cancer cell models. Prostate. 2019;79(1):21–30. 30106164 10.1002/pros.23707

[258] Gravina GL, Festuccia C, Millimaggi D, Dolo V, Tombolini V, de Vito M, Vicentini C, Bologna M. Chronic azacitidine treatment results in differentiating effects, sensitizes against bicalutamide in androgen-independent prostate cancer cells. Prostate. 2008;68(7):793–801. 18324645 10.1002/pros.20748

[259] Sonpavde G, Aparicio AM, Zhan F, North B, DeLaune R, Garbo LE, Rousey SR, Weinstein RE, Xiao L, Boehm KA, et al. Azacitidine favorably modulates PSA kinetics correlating with plasma DNA LINE-1 hypomethylation in men with chemonaive castration-resistant prostate cancer. Urol Oncol. 2011;29(6):682–689. 19959380 10.1016/j.urolonc.2009.09.015

[260] Lin D, Dong X, Wang K, Wyatt AW, Crea F, Xue H, Wang Y, Wu R, Bell RH, Haegert A, et al. Identification of DEK as a potential therapeutic target for neuroendocrine prostate cancer. Oncotarget. 2015;6(3):1806–1820. 25544761 10.18632/oncotarget.2809 PMC4359333

[261] Mor-Vaknin N, Saha A, Legendre M, Carmona-Rivera C, Amin MA, Rabquer BJ, Gonzales-Hernandez MJ, Jorns J, Mohan S, Yalavarthi S, et al. DEK-targeting DNA aptamers as therapeutics for inflammatory arthritis. Nat Commun. 2017;8:14252. 28165452 10.1038/ncomms14252 PMC5303823

